# CircCCNB1 inhibits vasculogenic mimicry by sequestering NF90 to promote miR‐15b‐5p and miR‐7‐1‐3p processing in nasopharyngeal carcinoma

**DOI:** 10.1002/1878-0261.13821

**Published:** 2025-02-18

**Authors:** Chunmei Fan, Fenghua Tan, Jie Wu, Zhaoyang Zeng, Wenjia Guo, He Huang, Wei Xiong

**Affiliations:** ^1^ NHC Key Laboratory of Carcinogenesis and Hunan Key Laboratory of Cancer Metabolism, Hunan Cancer Hospital and the Affiliated Cancer Hospital of Xiangya School of Medicine Central South University Changsha China; ^2^ Department of Histology and Embryology, Xiangya School of Medicine Central South University Changsha China; ^3^ Key Laboratory of Carcinogenesis and Cancer Invasion of the Chinese Ministry of Education, Cancer Research Institute Central South University Changsha China; ^4^ Department of Cancer Research Institute Affiliated Cancer Hospital of Xinjiang Medical University Urumqi China; ^5^ Xinjiang Key Laboratory of Translational Biomedical Engineering Urumqi China; ^6^ Furong Laboratory Changsha China

**Keywords:** circCCNB1, miRNA processing, nasopharyngeal carcinoma, NF90, vasculogenic mimicry

## Abstract

Nasopharyngeal carcinoma (NPC) is a kind of malignant tumor with high metastasis. Circular RNAs (circRNAs) are involved in tumor progression, but their functions and mechanisms are not well understood. Vasculogenic mimicry (VM) has been discovered as an alternative way to supply tumor nutrition and accelerate tumor progression, including NPC. We previously found that circCCNB1 (derived from cyclin B1) could inhibit the migration and invasion of NPC cells by binding to nuclear factor 90 (NF90), however, whether circCCNB1 has additional biological functions is still unclear. In this study, the effects of circCCNB1 binding to NF90 on the generation of miR‐15b‐5p and miR‐7‐1‐3p were detected using qRT‐PCR, western blotting, RNA pulldown, ribonucleoprotein immunoprecipitation and truncated experiments. VM formation assays were used to assess their biological functions. We found that circCCNB1 promoted the processing and generation of miR‐15b‐5p and miR‐7‐1‐3p through competitive binding to NF90, thereby inhibiting the expression of calumenin (CALU), kinesin family member 1B (KIF1B), RNA polymerase III subunit G (POLR3G), ultimately decreasing the VM of NPC cells. This study not only reveals a new function of circCCNB1 in NPC, but also provides new insights for targeting angiogenesis therapy.

## Graphical abstract

CircCCNB1 expression is down‐regulated in nasopharyngeal carcinoma (NPC); thus, less NF90 protein is bound to circCCNB1 and more binds to pri‐miRNAs, blocking their (pri‐miRNAs) binding to DGCR8 and inhibiting the processing and generation of miR‐15b‐5p/miR‐7‐1‐3p. Furthermore, decreased miR‐15b‐5p/miR‐7‐1‐3p promotes the expression of the target genes *KIF1B*,*CALU*, and *POLR3G*, thus elevating the expression of MMP9 and VE‐cadherin to promote the vasculogenic mimicry of NPC.

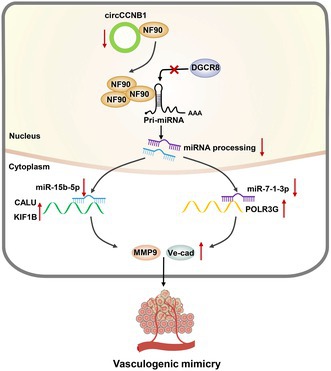

**Abbreviations**
CALU: calumenin
circRNA: circular RNA
DSRM: double‐stranded RNA‐binding motif
ILF3: interleukin enhancer binding factor 3
KIF1B: kinesin family member 1B
NF90: nuclear factor 90
NPC: nasopharyngeal carcinoma
POLR3G: RNA polymerase III subunit G
pri‐miRNA: primary miRNA
RIP: ribonucleoprotein immunoprecipitation
VM: vasculogenic mimicry

## 1 Introduction

Nasopharyngeal carcinoma (NPC) is a highly malignant tumor originating from the nasopharyngeal epithelium. It is prevalent in Southeast Asia, especially in the southern China [1]. Studies have shown that NPC is closely associated with genetic background, Epstein–Barr virus infection, and environmental factors, but the pathogenesis of NPC is far from being clarified [2]. As the onset site of NPC is hidden, and the early symptoms are not obvious, most patients are already at an advanced stage when diagnosed. Therefore, exploring the pathogenesis of NPC and seeking for potential therapeutic targets is of great significance for the treatment of this disease.

Circular RNAs (circRNAs) are non‐coding RNAs with covalently closed loop structures, which have been found to play important roles in the initiation and progression of malignant tumors in recent years. For example, circRNA can regulate tumor development by competitively adsorbing miRNAs to regulate the expression of target genes [3, 4], or by affecting epigenetic modification [5]. Some studies have shown that the stable presence of circRNA in exosomes induces chemotherapy resistance [6]. In addition, specific structures of circRNA can encode small peptides, making them suitable for the emerging circRNA vaccines [7]. At present, more than 30 000 circRNAs have been found, but the role of circRNAs in NPC is still unclear. We previously found that circRNA circCCNB1, formed by the back‐splicing of exons 6 and 7 of cyclin B1 (CCNB1) pre‐RNA, is significantly down‐regulated in NPC. CircCCNB1 binds to NF90 to inhibit migration and invasion of NPC [8]. As a multifunctional protein, NF90 plays a key role in cellular metabolic activities such as transcriptional activation, translation control, mRNA processing, and cell localization. However, whether circCCNB1 binding to NF90 plays other biological roles in NPC remains unclear.

Angiogenesis plays an important role in tumor growth, progression, and metastasis. As tumor grows, the lack of nutrients and oxygen inside the tumor triggers the up‐regulation of some pro‐angiogenic factors, leading to the formation of new blood vessels. Blood vessels not only provide nutritional support but also allow the exchange of information between primary tumors and metastases. Therefore, angiogenesis is important for tumor progression and is also a potential target for tumor therapy. However, the clinical efficacy of anti‐angiogenic drugs has been far below expectations, as the underlying mechanisms of angiogenesis have not been elucidated. Notably, Maniotis et al. discovered that cancer cells can form the tubular structures themselves to supply blood to tumor, a process termed as vasculogenic mimicry (VM), which may in part explain the failure of anti‐angiogenic drugs to prevent metastasis [9]. VM has been reported in several tumors such as glioma, ovarian cancer, and hepatocellular carcinoma [10]. However, VM formation and its regulatory mechanisms are far from clear in NPC.

In this study, we found that circCCNB1 promoted the processing and generation of miR‐7‐1‐3p and miR‐15b‐5p by binding to NF90, thereby inhibiting VM in NPC. Furthermore, circCCNB1 may become a potential therapeutic target for anti‐angiogenic therapy in NPC.

## 2 Materials and methods

### 2.1 Cell culture and transfection

The NPC cell lines HNE2 (RRID:CVCL_FA07) and CNE2 (RRID:CVCL_6889), were obtained from the Cell Culture Center of Central South University. The cells were cultured in RPMI 1640 medium (Gibco, Grand Island, NY, USA) supplemented with 10% FBS (Gibco) plus 1% penicillin/streptomycin in an incubator at 37 °C and 5% CO_2_. All cell lines were authenticated based on Short Tandem Repeat (STR) technique in the past 3 years. All experiments were performed with mycoplasma‐free cells.

The circCCNB1 overexpression plasmid, containing exons 6 and 7 of CCNB1 pre‐RNA was cloned into pcDNA3.1(+) CircRNA Mini Vector (the empty vector is a gift from Y. Li, Baylor College of Medicine). pCMV3‐NF90‐Flag construct was purchased from Sino Biological Corporation (Beijing, China). siRNAs were purchased from RiboBio Co., Ltd (Guangzhou, China). The siRNA sequences are shown in Table S1. For plasmids transfection, Neofect transfection reagent (Neofect biotech Co., Ltd., Beijing, China) was used; for transfection of siRNAs, miRNA mimics or inhibitors, Hiperfect transfection reagent (Qiagen, Hilden, Germany) was employed as per the manufacturer's instructions.

### 2.2 RNA extraction, reverse transcription, and quantitative real‐time PCR

Total RNA extraction was performed using TRIzol reagent (Life, Grand Island, NY, USA) and cDNA samples were obtained using HiScript II Q RT SuperMix for qPCR kit (Vazyme, Nanjing, China) according to the manufacturer's instructions. Stem‐loop qRT‐PCR for mature miRNAs was performed with the Qiagen QuantiTect SYBR Green PCR Kits (Qiagen). Quantitative real‐time PCR (qRT‐PCR) for RNA expression was performed using the 2× SYBR Green qPCR Master Mix kit (Bimake, Houston, TX, USA). qRT‐PCR amplification reaction was run on CFX Real‐Time PCR Detection System with cfx manager™ software, version 3.1 (Bio‐Rad, Hercules, CA, USA). Relative RNA expression was normalized by GAPDH using $2^{-\Delta \Delta C_{t}}$ quantification method. The sequences of qRT‐PCR primers are listed in Table S2.

### 2.3 Western blotting

RIPA (Beyotime Biotechnology, Shanghai, China) and protease inhibitor cocktail (Roche Applied Sciences, Mannheim, Germany) were used for protein extraction. The concentration of protein samples was evaluated using the BCA assay kit (Bio‐Rad). In general, 30–50 μg denatured protein was separated by 10% SDS/PAGE and transferred to a 0.2 μm PVDF membrane (Millipore, Billerica, MA, USA). The PVDF membrane was blocked with 5% skimmed milk at room temperature for 1 h, incubated with primary antibody at 4 °C overnight, washed with 1× PBST for three times, and then incubated with peroxidase‐conjugated secondary antibody at room temperature for 2 h. After washing with 1× PBST for three times, the PVDF membrane was developed using the eECL Western Blot Kit (CWBIO Technology, Taizhou, China) and imaged by ECL detection system (Millipore). The antibodies used are listed in Table S3.

### 2.4 Co‐immunoprecipitation (Co‐IP)

After transfection for 48 h, the cells were lysed with GLB^+^ buffer (10 mm NaCl, 10 mm EDTA, 10 mm Tris/HCl, 0.5% Triton‐100) containing a proteinase inhibitor cocktail (Keygen, Nanjing, China). Cell lysates were incubated with 1 μg anti‐NF90 (or negative control IgG) and 50 μL protein A/G magnetic beads (Bimake) overnight at 4 °C with rotation. After washing with GLB^+^ buffer for 5–6 times, the precipitates were analyzed by western blotting using anti‐DGCR8 or anti‐Drosha antibodies.

### 2.5 RNA pulldown

The circCCNB1 probe was transfected into the cells, after 48 h, the cells were lysed with ribonucleoprotein immunoprecipitation (RIP) buffer (150 mm KCl, 25 mm Tris–HCl, 0.5 mm DTT, 0.5% NP40). Fifty microliter Streptavidin Dynabeads (M‐280; Invitrogen, Carlsbad, CA, USA) was added into the lysates for overnight incubation at 4 °C with rotation. The next day, the precipitates were washed 6 times with RIP buffer, and then subjected to western blotting.

### 2.6 Ribonucleoprotein immunoprecipitation

Ribonucleoprotein immunoprecipitation was performed using the Magna RIP TM Kit (17‐701; Millipore) according to the manufacturer's instruction. Briefly, cells were lysed using the RIP lysis buffer containing a protease inhibitor cocktail (Keygen). Magnetic beads were washed twice with RIP wash buffer, incubated with 1–5 μg anti‐NF90, anti‐Flag, anti‐DGCR8 or IgG control antibody, and rotated slowly at room temperature for 30 min. Next, 100 μL cell lysates were mixed with 900 μL RIP immunoprecipitation buffer, subsequently incubated with magnetic beads overnight at 4 °C. After washing the immunoprecipitation six times with RIP wash buffer, protein K buffer was added and incubated at 55 °C for 30 min, followed by phenol‐chloroform extraction. The immunoprecipitated RNA was extracted using the TRIzol reagent and subjected to qRT‐PCR to test RNA enrichment.

### 2.7 Vasculogenic mimicry experiment

To observe VM, NPC cells were subjected to tube formation assays. Briefly, 50 μL matrigel was plated in the 96‐well plate, and then the plate was placed at 37 °C for 1 h to allow matrigel polymerization. The transfected NPC cells (CNE2 or HNE2) were diluted to 3 × 10^4^ cells/well, and the cells were added to the 96‐well plate, which was laid on matrigel and cultured at 37 °C for 4–6 h. Randomized fields were captured under an inverted phase contrast microscope. The number of tubes was counted using imagej software (Media Cybernetics, Rockville, MD, USA). The data are presented as the average numbers of tubes ± standard deviation.

### 2.8 Dual‐luciferase reporter assay

For CALU or KIF1B, the CNE2 or HNE2 cells were co‐transfected with miR‐15b‐5p mimics or inhibitors, the luciferase reporter vector (CALU‐WT, CALU‐MT or KIF1B‐WT, KIF1B‐MT) and the pRL‐TK Renilla luciferase vector (Promega, Madison, WI, USA). For POLR3G, the CNE2 or HNE2 cells were co‐transfected with miR‐7‐1‐3p mimics or inhibitors, the luciferase reporter vector (POLR3G‐WT or POLR3G‐MT) and the pRL‐TK Renilla luciferase vector. Luciferase activity was measured using the Dual‐Luciferase® Reporter Assay System (E1910; Promega) 48 h after transfection by plate reader with softmax® Pro 7 software (Molecular Devices, Shanghai, China), version 7.1.0. Relative luciferase activity was normalized to Renilla activity. The synthetic wild‐type and mutant sequences are shown in Table S4.

### 2.9 Data analysis

Statistical analysis was performed using graphpad prism 9.0 software (GraphPad, La Jolla, CA, USA). Student's *t* test was used to analyze the significance of data. All data were represented as mean ± standard deviation (SD), and *P* < 0.05 was considered significant (*P < 0.05, **P < 0.01, ***P < 0.001, ****P < 0.0001).

## 3 Results

### 3.1 CircCCNB1 promotes the processing and generation of miR‐15b‐5p and miR‐7‐1‐3p via NF90

**Fig. 1:**
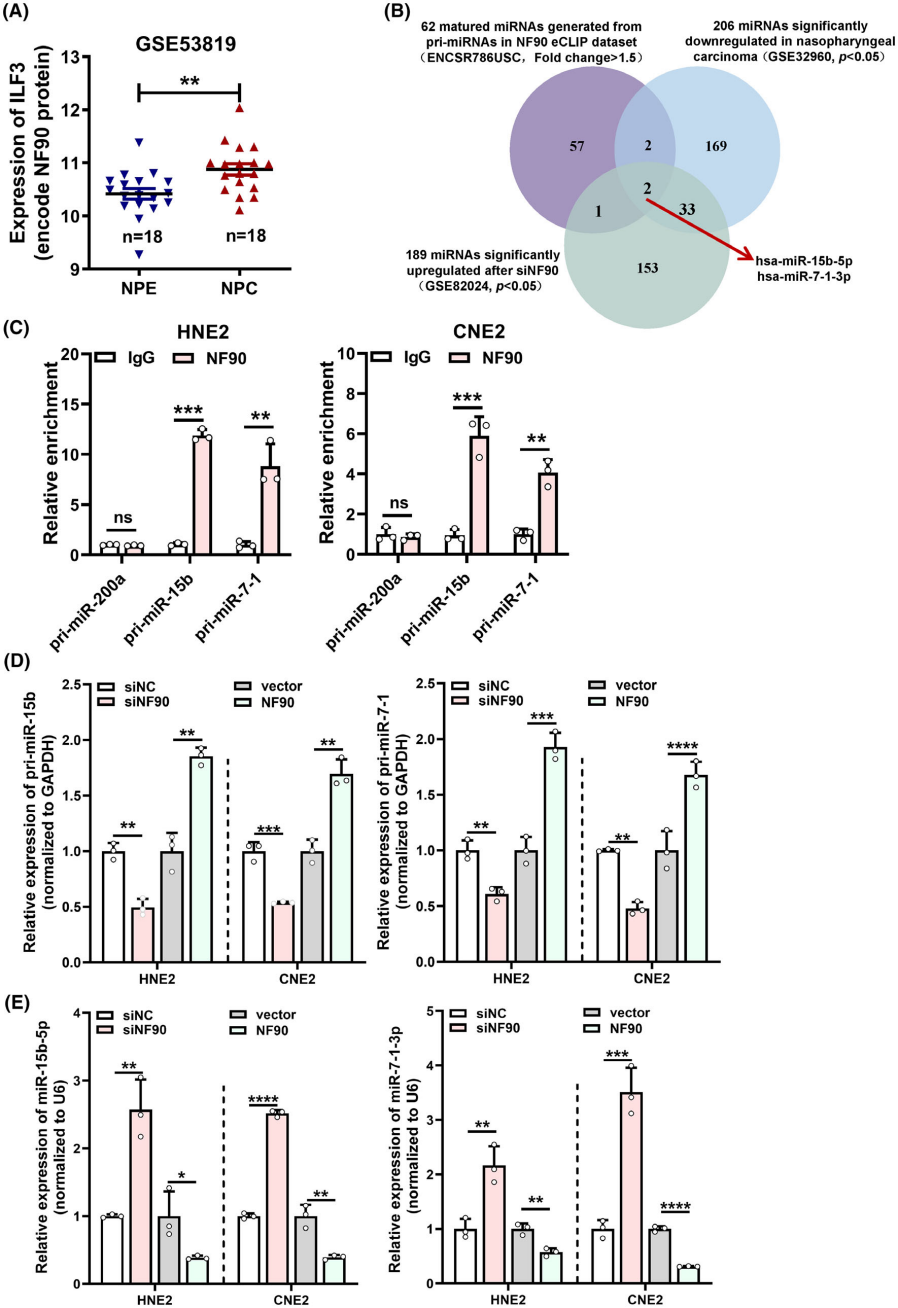
NF90 inhibits processing and generation of miR‐15b‐5p and miR‐7‐1‐3p. (A) Expression of ILF3 (encode NF90 protein) in nasopharyngeal carcinoma (NPC) tissues and normal nasopharyngeal epithelium (NPE) from microarray GSE53819. (B) NF90 eCLIP dataset (ENCSR786USC), up‐regulated miRNAs after siNF90 (GSE82024) and down‐regulated miRNAs in NPC (GSE32960) were intersected. (C) Ribonucleoprotein immunoprecipitation assays were performed to detect the enrichment of anti‐NF90 for pri‐miR‐15b and pri‐miR‐7‐1. (D, E) qRT‐PCR experiments were employed to measure the expression of pri‐miR‐15b and pri‐miR‐7‐1 or their mature miRNAs in HNE2 and CNE2 cells after overexpression or knockdown of NF90. siNF90:siRNA targeting NF90. Unpaired two‐tailed Student's *t* test was used for (C–E), *P < 0.05, **P < 0.01, ***P < 0.001, and ****P < 0.0001; ns, not significant. These experiments were derived from three independent repetitions. Data are presented as mean ± SD.

NF90 is highly expressed in various tumors. To explore the potential role of NF90 in NPC, we first analyzed ILF3 (Interleukin Enhancer Binding Factor 3, encoding NF90 protein) expression through GSE53819. GSE53819 is a genome‐wide expression profile containing 18 NPC tissues versus 18 non‐cancerous nasopharyngeal tissues. The results showed that ILF3 is highly expressed in NPC tissue, suggesting its possible oncogenic role in NPC (Fig. 1A), which is also up‐regulated in head and neck squamous cell carcinoma (HNSC) tissues compared with normal tissues in TCGA database (Fig. S1A). NF90 plays a critical role in transcriptional activation, translational control, mRNA processing, and cellular localization. Additionally, we noticed that NF90 may inhibit miRNA processing [11]. In order to screen miRNAs that might be regulated by NF90, we downloaded the eCLIP dataset of NF90 (ENCSR786USC) and identified 38 primary miRNAs (pri‐miRNAs) potentially interacting with NF90 (Fold Change > 1.5). These pri‐miRNAs generate 62 mature miRNAs (Table S5). Considering NF90 is negative related with miRNA generation, we selected the up‐regulated miRNAs after siNF90 from GSE82024. Furthermore, due to the up‐regulation of NF90 and the negative regulation of miRNAs in NPC, we selected the down‐regulated miRNAs in NPC microarray profile GSE32960. By intersecting these three datasets, we finally identified hsa‐miR‐15b‐5p and hsa‐miR‐7‐1‐3p as potential miRNAs regulated by NF90 (Fig. 1B). Analysis of the GSE32960 microarray data revealed that miR‐15b‐5p and miR‐7‐1‐3p were indeed down‐regulated in NPC tissues (Fig. S1B).

The stem‐loop structures of pri‐miRNAs bound by NF90 contain fewer mismatches and longer double‐stranded regions, and are enriched with A/U sequences [12]. We used RNAfold to predict secondary structures of pri‐miRNAs. The results revealed that pri‐miR‐15b and pri‐miR‐7‐1 have more A/U‐rich sequences and fewer mismatches compared to the negative control pri‐miR‐200a from the eCLIP data (Fig. S1C), consistent with the characteristics of NF90‐bound pri‐miRNAs. Subsequently, through RIP assays, we found that anti‐NF90 could enrich more pri‐miR‐15b and pri‐miR‐7‐1, while there was no significant enrichment for pri‐miR‐200a (negative control) (Fig. 1C). To investigate the impact of NF90 binding on the expression of pri‐miR‐15b, pri‐miR‐7‐1, as well as mature miR‐15b‐5p and miR‐7‐1‐3p, we performed overexpression or knockdown of NF90 and tested NF90 expression by qRT‐PCR and western blotting. The results showed that NF90 is successfully knocked down or overexpressed (Fig. S1D,E). Furthermore, it was observed that overexpression of NF90 promoted pri‐miR‐15b and pri‐miR‐7‐1 expression but reduced mature miR‐15b‐5p and miR‐7‐1‐3p levels, and vice versa (Fig. 1D,E). These results indicated that NF90 binds to pri‐miR‐15b and pri‐miR‐7‐1, leading to their processing inhibition, thereby suppressing the generation of mature miR‐15b‐5p and miR‐7‐1‐3p.

**Fig. 2:**
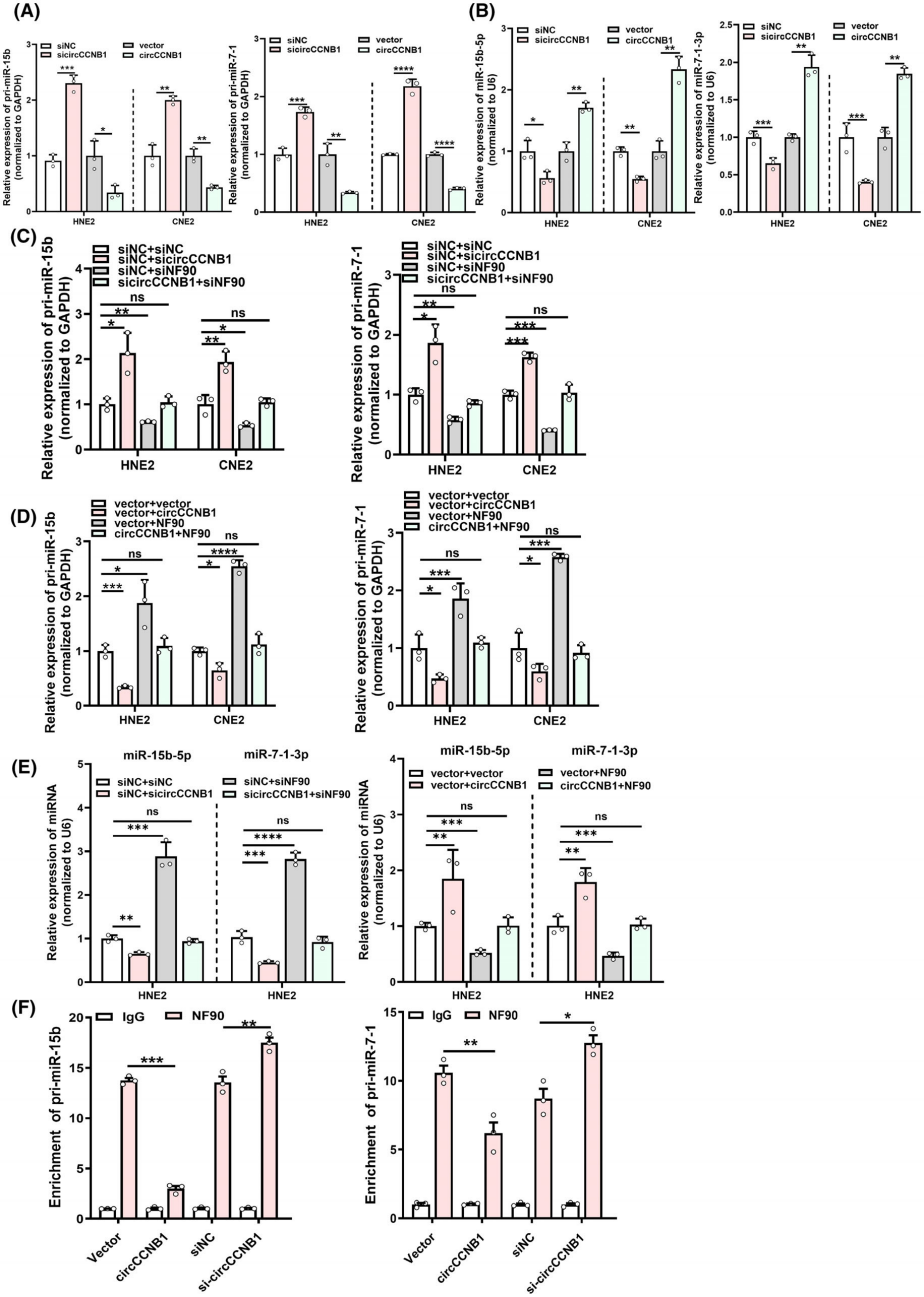
CircCCNB1 promotes the processing and generation of miR‐15b‐5p and miR‐7‐1‐3p via NF90. (A, B) qRT‐PCR experiments were performed to measure the expression of pri‐miR‐15b and pri‐miR‐7‐1 or mature miR‐15b‐5p and miR‐7‐1‐3p after overexpression or knockdown of circCCNB1. (C, D) qRT‐PCR experiments were conducted to measure pri‐miR‐15b and pri‐miR‐7‐1 expression after simultaneous knockdown of circCCNB1 and NF90 or simultaneous overexpression of circCCNB1 and NF90. (E) qRT‐PCR experiments were employed to measure miR‐15b‐5p and miR‐7‐1‐3p levels after simultaneous knockdown of circCCNB1 and NF90 (left) or simultaneous overexpression of circCCNB1 and NF90 (right). (F) Ribonucleoprotein immunoprecipitation assays were used to detect the enrichment of pri‐miR‐15 and pri‐miR‐7‐1 by anti‐NF90 after overexpression or knockdown of circCCNB1. Unpaired two‐tailed Student's *t* test was used for (A–F), *P < 0.05, **P < 0.01, ***P < 0.001, and ****P < 0.0001; ns, not significant. These experiments were derived from three independent repetitions. Data are presented as mean ± SD.

We previously reported that circCCNB1 binds to NF90 and inhibits migration and invasion in NPC. However, it is unclear whether circCCNB1 binding to NF90 exerts other biological functions, especially in regulating miRNA generation. To investigate whether circCCNB1 affects miRNA generation, we performed qRT‐PCR and found that overexpression of circCCNB1 inhibits pri‐miR‐15b and pri‐miR‐7‐1 expression but promotes mature miR‐15b‐5p and miR‐7‐1‐3p levels (Fig. 2A,B), which is opposite to the regulation by NF90. To confirm whether the effect of circCCNB1 on miRNA generation is mediated via NF90, we simultaneously knocked down circCCNB1 and NF90 to perform rescue experiments. The results showed that silencing circCCNB1 and NF90 simultaneously partially reverses the promoting effect of sicircCCNB1 on pri‐miR‐15b and pri‐miR‐7‐1 expression (Fig. 2C), while overexpressing circCCNB1 and NF90 simultaneously, NF90 partially reverses the inhibitory effect of circCCNB1 on pri‐miR‐15b and pri‐miR‐7‐1 expression (Fig. 2D). Furthermore, we conducted rescue experiments to examine the effects of circCCNB1 and NF90 on mature miRNAs. When cells were co‐transfected with sicircCCNB1 and siNF90, siNF90 partially reversed the inhibitory effect of sicircCCNB1 on miR‐15b‐5p and miR‐7‐1‐3p, and vice versa (Fig. 2E). RIP assays revealed that overexpression of circCCNB1 reduces the binding of NF90 to pri‐miR‐15b and pri‐miR‐7‐1, while knockdown of circCCNB1 increases the binding of NF90 to pri‐miR‐15b and pri‐miR‐7‐1 (Fig. 2F). These results suggest that circCCNB1 regulates the processing and generation of miR‐15b‐5p and miR‐7‐1‐3p via NF90.

### 3.2 CircCCNB1 binds to the DSRM2 domain of NF90 via its stem‐loop 1

**Fig. 3:**
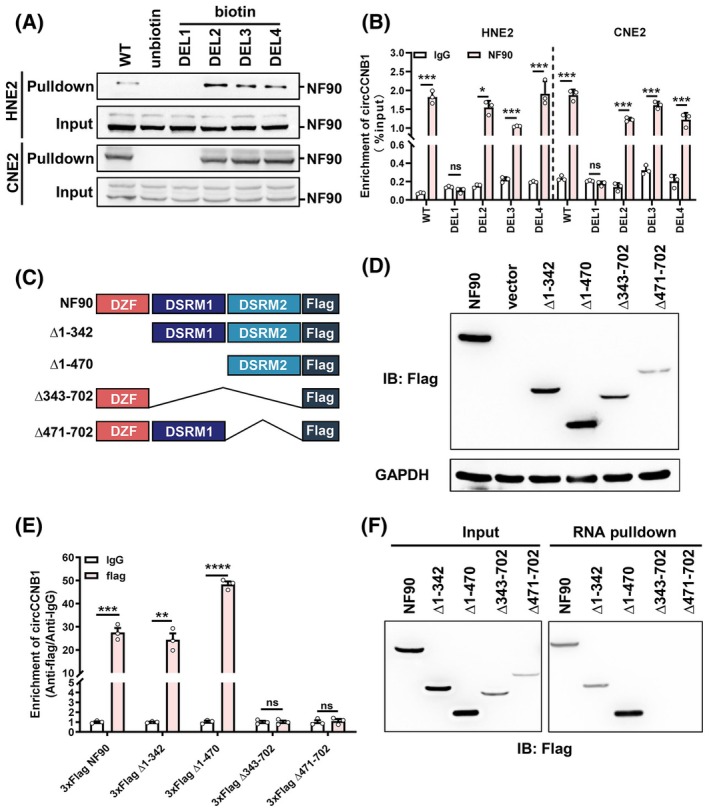
circCCNB1 binds to the DSRM2 domain of NF90 through its stem‐loop 1. (A) RNA pulldown combined with western blotting experiments were used to detect the binding between circCCNB1 mutants and NF90. (B) Ribonucleoprotein immunoprecipitation combined with qRT‐PCR experiments were employed to detect the enrichment of anti‐NF90 for circCCNB1 by overexpressing different circCCNB1 mutants. (C) Schematic diagram of NF90 wild‐type and truncated mutants. (D) The expression of Flag‐tagged NF90 truncated mutants were detected using western blotting. (E) Ribonucleoprotein immunoprecipitation combined with qRT‐PCR experiments were conducted to detect the enrichment of circCCNB1 by anti‐NF90 after overexpressing NF90 truncated mutants. (F) The binding ability between circCCNB1 and NF90 mutants were detected using RNA pulldown. Unpaired two‐tailed Student's *t* test was used for (B, E), *P < 0.05, **P < 0.01, ***P < 0.001, and ****P < 0.0001; ns, not significant. qPCR experiments were derived from three independent repetitions. Each western blots were reproduced three times with similar results. Data are presented as mean ± SD.

Through circRNADb prediction, circCCNB1 could not translate to small peptide as no open reading frame was found (Fig. S2A), and it did not regulate NF90 expression (Fig. S2B). To further explore the specific binding site between circCCNB1 and NF90, according to Lingling Chen's research, compared to linear RNAs, circRNAs have more stem‐loop structures which play a crucial role in circRNA‐protein interactions [13]. To identify whether the stem‐loop structure of circCCNB1 affects its binding to NF90, we used RNAfold software to predict the secondary structure of circCCNB1 and found that it mainly consists of four prominent stem‐loop (Fig. S2C). Using catRAPID, we predicted the binding region between circCCNB1 and NF90 and found that these four stem‐loop of circCCNB1 may be the regions binding to NF90 (Fig. S2D). We predicted the sequences with deletion of each of the four different stem‐loop using RNAfold and observed that the corresponding stem‐loop disappeared upon deletion. We constructed mutants of circCCNB1 with deletions in these four stem‐loop regions respectively (Fig. S2E). RNA pulldown and RIP assays indicated that circCCNB1 DEL1 cannot bind to NF90, suggesting that NF90 binds to loop 1 of circCCNB1 (Fig. 3A,B). By querying the UniProt database, we found that NF90 possesses three domains: zinc finger domain (DZF), double‐stranded RNA‐binding motif 1 (DSRM1), and double‐stranded RNA‐binding motif 2 (DSRM2). We constructed different truncated NF90 constructs tagged with Flag and verified the expression using anti‐Flag antibody. The results showed that the truncated mutants correctly expressed the Flag tag (Fig. 3C,D). RIP and RNA pulldown assays revealed that the mutants ∆343–702 and ∆471–702 (both lacking the DSRM2 domain) completely disrupted the binding between circCCNB1 and NF90, while deletion of the DZF and DSRM1 domains had little effect on the binding ability. These results suggest that circCCNB1 binds to the DSRM2 domain of NF90 (Fig. 3E,F).

### 3.3 CircCCNB1 competitively binds to NF90 and promotes the processing of pri‐miR‐15b‐5p and pri‐miR‐7‐1‐3p by DGCR8

**Fig. 4:**
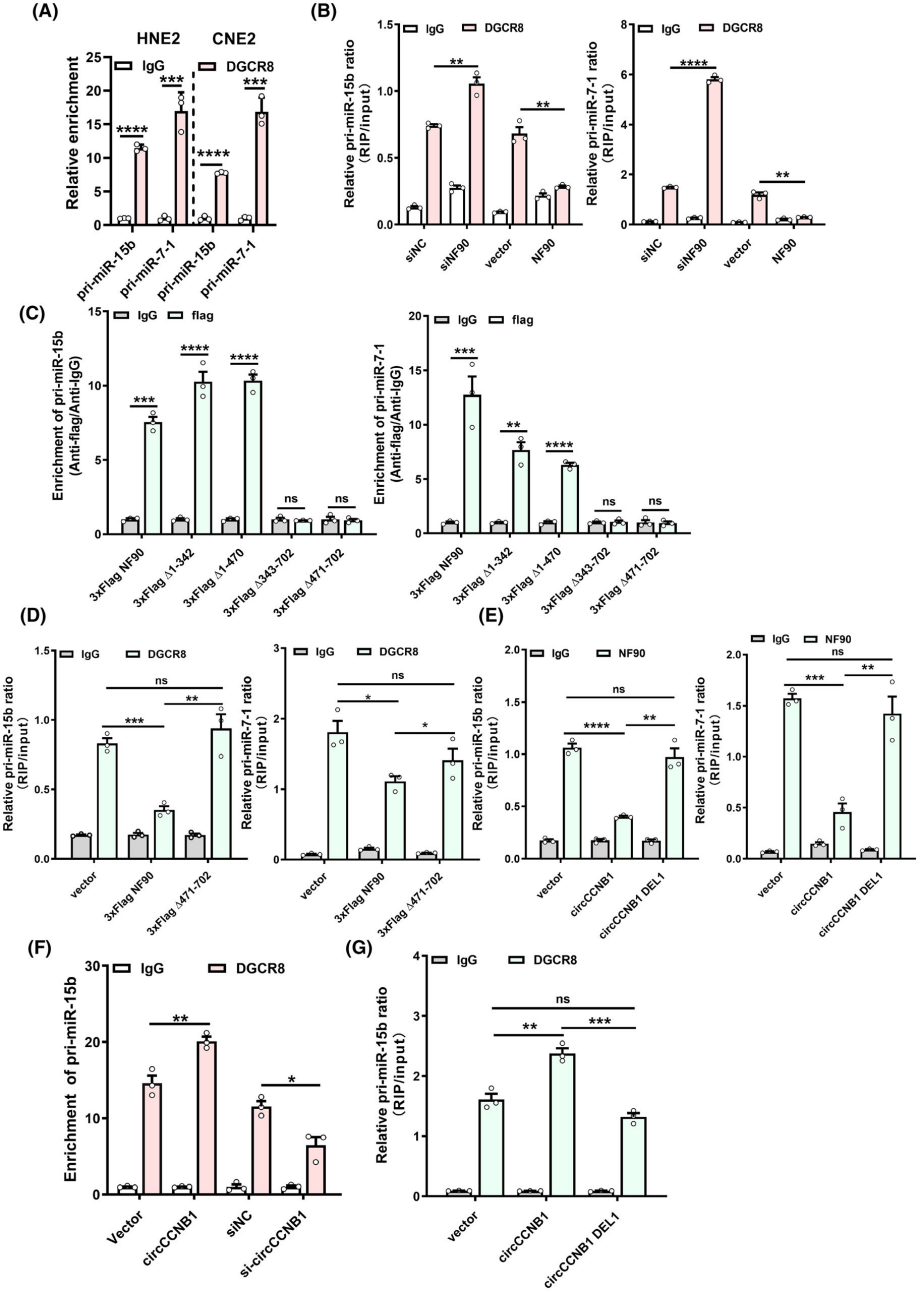
CircCCNB1 competitively interacts with NF90, promoting DGCR8‐mediated processing of pri‐miR‐15b‐5p and pri‐miR‐7‐1‐3p. (A) The enrichment of pri‐miR‐15b and pri‐miR‐7‐1 by anti‐DGCR8 was detected using RIP assays. (B) RIP assays were performed to examine the enrichment of pri‐miR‐15b and pri‐miR‐7‐1 by anti‐DGCR8 after overexpression or knockdown of NF90. (C) RIP assays were employed to assess the enrichment of pri‐miR‐15b and pri‐miR‐7‐1 by anti‐Flag after overexpression of NF90 wild‐type and mutants. (D) RIP assays were used to determine the binding of DGCR8 to pri‐miR‐15b and pri‐miR‐7‐1 after overexpression of wild‐type NF90 and DSRM2 mutant. (E) RIP assays were used to evaluate the binding of NF90 to pri‐miR‐15b and pri‐miR‐7‐1 after overexpression of circCCNB1 and circCCNB1 DEL1 mutant. (F) The binding ability between DGCR8 and pri‐miR‐15b was examined using RIP after overexpression or knockdown of circCCNB1. (G) The binding between DGCR8 and pri‐miR‐15b was evaluated using RIP after overexpression of circCCNB1 and circCCNB1 DEL1 mutant. RIP, ribonucleoprotein immunoprecipitation. Unpaired two‐tailed Student's *t* test was used for (A–G), *P < 0.05, **P < 0.01, ***P < 0.001, and ****P < 0.0001; ns, not significant. These experiments were derived from three independent repetitions. Data are presented as mean ± SD.

DGCR8 is a key enzyme in miRNA processing by forming microprocessor complex. To explore whether DGCR8 is involved in the processing of pri‐miR‐15b and pri‐miR‐7‐1, we conducted RIP assays and found that DGCR8 binds to pri‐miR‐15b and pri‐miR‐7‐1 (Fig. 4A), indicating the involvement of the DGCR8 microprocessor complex in the processing of pri‐miR‐15b and pri‐miR‐7‐1.

To further investigate the specific mechanism of circCCNB1 binding to NF90 in regulating miRNA processing, we conducted RNA pulldown and co‐IP assays. We found that neither DGCR8 nor Drosha binds to circCCNB1 or NF90 (Fig. S3A,B). This result suggests that circCCNB1 does not directly rely on the microprocessor proteins for miRNA processing. However, when NF90 is overexpressed, the binding of DGCR8 to pri‐miR‐15b and pri‐miR‐7‐1 decreases. Conversely, when NF90 is knocked down, the binding of DGCR8 to pri‐miR‐15b and pri‐miR‐7‐1 increases (Fig. 4B). This phenomenon indicates that NF90 may compete with DGCR8 on binding to pri‐miR‐15b and pri‐miR‐7‐1, leading to processing inhibition. In addition, when different NF90 truncated mutants were transfected into NPC cells and subjected to RIP assays, it was found that when the DSRM2 domain is deleted, NF90 cannot bind to pri‐miR‐15b and pri‐miR‐7‐1. However, deletion of other domains has little impact on the binding of NF90 to both two pri‐miRNAs (Fig. 4C). Furthermore, when the DSRM2 domain of NF90 is deleted, as NF90 cannot bind to pri‐miR‐15b and pri‐miR‐7‐1, the competitive effect between NF90 and DGCR8 disappears (Fig. 4D). These results further indicate that NF90 competitively binds to pri‐miR‐15b and pri‐miR‐7‐1 through its DSRM2 domain, thereby inhibiting the binding of DGCR8 to pri‐miRNAs.

To explore the role of circCCNB1 in this process, we found that overexpression of circCCNB1 leads to a decrease in the binding of NF90 to pri‐miR‐15b and pri‐miR‐7‐1. However, when loop 1 of circCCNB1 is deleted (DEL1), the expression of pri‐miR‐15b and pri‐miR‐7‐1 is restored, indicating that circCCNB1 regulates the binding of NF90 to pri‐miR‐15b and pri‐miR‐7‐1 via its loop 1 (Fig. 4E). Through RIP assays, we found that overexpression of circCCNB1 increases the binding of DGCR8 to pri‐miR‐15b and pri‐miR‐7‐1, while knockdown of circCCNB1 decreases the binding effects (Fig. 4F, Fig. S3C). When loop 1 of circCCNB1 is deleted, it partially counteracts the increased binding of DGCR8 to pri‐miR‐15b and pri‐miR‐7‐1 caused by overexpressing circCCNB1, suggesting that the loop 1 region of circCCNB1 regulates the binding of DGCR8 to pri‐miR‐15b and pri‐miR‐7‐1 (Fig. 4G, Fig. S3D). We then examined the effect of circCCNB1 and its mutant DEL1 on the expression of pri‐miRNAs and mature miRNAs. We found that overexpression of circCCNB1 leads to a decrease in the level of pri‐miR‐15b and pri‐miR‐7‐1, while increasing the level of their mature forms. However, when circCCNB1 DEL1 is overexpressed, this phenomenon is restored (Fig. S3E,F). In summary, these results indicate that circCCNB1 competitively binds to the DSRM2 domain of NF90 via its loop 1 region, preventing NF90 from binding to pri‐miR‐15b and pri‐miR‐7‐1, which promotes the binding of DGCR8 to pri‐miR‐15b and pri‐miR‐7‐1, leading to enhanced processing and generation of mature miR‐15b‐5p and miR‐7‐1‐3p.

### 3.4 miR‐15b‐5p and miR‐7‐1‐3p target CALU/KIF1B and POLR3G respectively

**Fig. 5:**
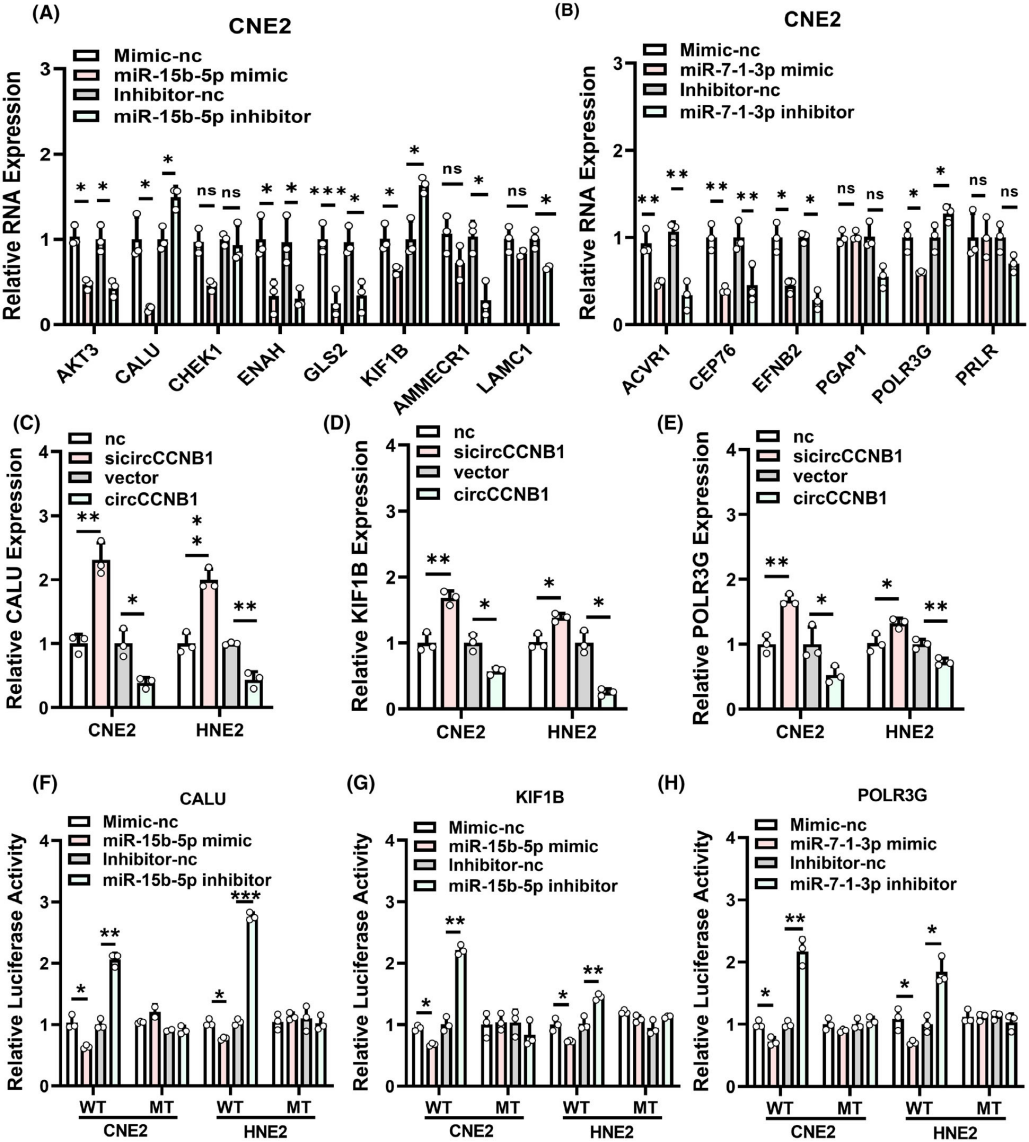
miR‐15b‐5p and miR‐7‐1‐3p target CALU/KIF1B and POLR3G, respectively. (A, B). qRT‐PCR was performed to measure the expression of downstream genes in CNE2 cells transfected with miR‐15b‐5p/miR‐7‐1‐3p mimic or inhibitor. (C–E). The effect of knockdown or overexpression of circCCNB1 on CALU, KIF1B, POLR3G levels was assessed using qRT‐PCR. (F, G). Luciferase reporter assay was performed to measure the luciferase activity of CALU, KIF1B wild‐type and mutant when co‐transfected with miR‐15b‐5p mimic or inhibitor. (H). Luciferase reporter assay was performed to measure the luciferase activity of POLR3G wild‐type and mutant when co‐transfected with miR‐7‐1‐3p mimic or inhibitor. Unpaired two‐tailed Student's *t* test was used for (A–H), *P < 0.05, **P < 0.01, ***P < 0.001; ns, not significant. These experiments were derived from three independent repetitions. Data are presented as mean ± SD.

Since the usual function of miRNAs is to bind to the 3′UTR of target mRNA, thus leading to degradation of the target mRNA or translation inhibition. We predicted the target genes of miR‐15b‐5p and miR‐7‐1‐3p using miRDIP, Targetscan, miRdb, and miRwalk software. Due to the down‐regulation of miR‐15b‐5p and miR‐7‐1‐3p in NPC, we selected up‐regulated mRNAs from the NPC datasets GSE12452 and GSE53819 and intersected them with the predicted target genes of miR‐15b‐5p or miR‐7‐1‐3p. As a result, 8 target genes including AKT3, CALU, CHEK1, ENAH, GLS2, KIF1B, AMMECR1, LAMC1 were potential targets of miR‐15b‐5p and 6 target genes including ACVR1, CEP76, EFNB2, PGAP1, POLR3G, PRLR were possibly candidates for miR‐7‐1‐3p (Fig. S4A,B). By performing qRT‐PCR experiments after cells transfected with miR‐15b‐5p/miR‐7‐1‐3p mimics or inhibitors, we found that calumenin (CALU) and kinesin family member 1B (KIF1B) were negatively regulated by miR‐15b‐5p, and RNA polymerase III subunit G (POLR3G) was negatively regulated by miR‐7‐1‐3p, suggesting that CALU/KIF1B and POLR3G are potential target genes (Fig. 5A,B, Fig. S4C,D). The same effects were achieved by western blotting (Fig. S4E,F). We then analyzed CALU, KIF1B, and POLR3G expression in TCGA database and NPC microarray GSE53819, the results showed that CALU and POLR3G are highly expressed in HNSC tumor tissues while KIF1B is not significant (Fig. S5A), but all of them are highly expressed in NPC tissues in GSE53819 (Fig. S5B). Furthermore, we observed that circCCNB1 could negatively regulate the expression of CALU/KIF1B and POLR3G (Fig. 5C–E). To determine the specific binding sites of miR‐15b‐5p and miR‐7‐1‐3p with their target genes, we used RNAhybrid website to predict their binding positions based on minimum free energy and complementary base pairing. We constructed wild‐type and mutant constructs for CALU/KIF1B and POLR3G (Fig. S5C–E) and confirmed that miR‐15b‐5p targets CALU/KIF1B and miR‐7‐1‐3p targets POLR3G through luciferase reporter assays (Fig. 5F–H).

### 3.5 The circCCNB1‐NF90‐miR‐15b‐5p/miR‐7‐1‐3p axis inhibits the expression of CALU/KIF1B or POLR3G, thereby suppressing VM in NPC cells

Studies have shown that miR‐15b‐5p has an inhibitory effect on angiogenesis [14, 15, 16, 17]. One of the target genes of miR‐15b‐5p, KIF1B, is a motor protein involved in mitochondrial transport. KIF1B positively regulates the expression of MT1‐MMP (membrane type 1‐matrix metalloproteinase), which promotes tumor metastasis by affecting processes such as extracellular matrix remodeling and angiogenesis [18, 19]. Another target gene of miR‐15b‐5p, CALU, is a calcium‐binding protein that protects fibulin‐1 from cleavage by MMP‐13. CALU is also associated with angiogenesis [20]. The target gene of miR‐7‐1‐3p, POLR3G, is regulated by the transcription factor MYC and is highly expressed in embryonic stem cells and some tumor cells [21]. POLR3G can regulate the stemness of lung cancer [22] and the metastasis of triple‐negative breast cancer [23]. The common pathways shared by these downstream molecules suggest angiogenesis, indicating that CALU/KIF1B or POLR3G may collectively target the angiogenesis pathway in tumors.

VM is a newly discovered alternative way of angiogenesis. An important reason for the failure of many anti‐angiogenic drugs is the presence of VM within the tumor [24]. While existing literature has been reported that NF90 regulates the angiogenesis process in endothelial cells [25, 26], there is limited research on the role of NF90 in tumor cell‐mediated VM formation. Moreover, there is a scarcity of reports specifically addressing VM in NPC cells. Therefore, we focus on investigating whether the interaction between circCCNB1 and NF90 affects VM in NPC.

**Fig. 6:**
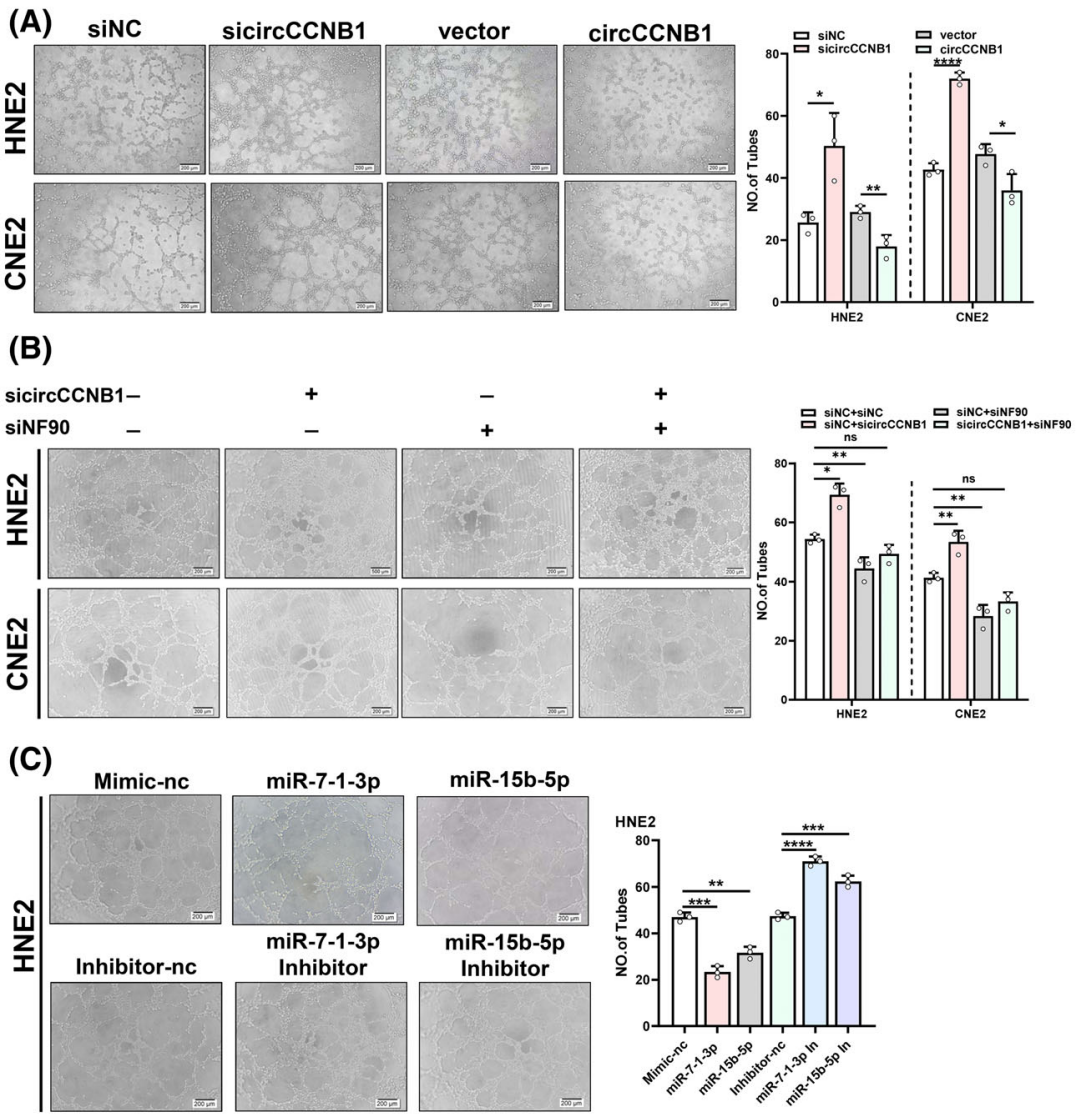
Roles of circCCNB1, NF90, miR‐15b‐5p/miR‐7‐1‐3p in vasculogenic mimicry in NPC cells. (A) After knockdown or overexpression of circCCNB1 in CNE2 and HNE2, the VM formation was detected by tube formation assay. Scale bars = 200 μm. (B) Co‐transfected sicircCCNB1 and siNF90, the tube formation ability was detected by tube formation assay. Scale bars = 200 μm. (C) HNE2 cells transfected with miR‐15b‐5p/miR‐7‐1‐3p mimics or inhibitors, tube formation assay was employed to evaluate VM. Scale bars = 200 μm. VM, vasculogenic mimicry. Unpaired two‐tailed Student's *t* test was used for (A–C), *P < 0.05, **P < 0.01, ***P < 0.001, and ****P < 0.0001; ns, not significant. These experiments were derived from three independent repetitions. Data are presented as mean ± SD.

To investigate whether circCCNB1 is involved in VM in NPC cells, we conducted tube formation assays to test VM formation. We found that knocking down circCCNB1 in NPC cells enhanced the tube formation ability, while overexpressing circCCNB1 inhibited the formation of VM in the cells (Fig. 6A). Simultaneous knocking down of circCCNB1 and NF90 weakened the pro‐angiogenic effect caused by sicircCCNB1, and vice versa (Fig. 6B), while simultaneous overexpression of circCCNB1 and NF90 relieved the inhibitory effect of circCCNB1 on tube formation (Fig. S6A). These findings indicate that circCCNB1 inhibits the formation of VM in NPC cells by binding to NF90.

**Fig. 7:**
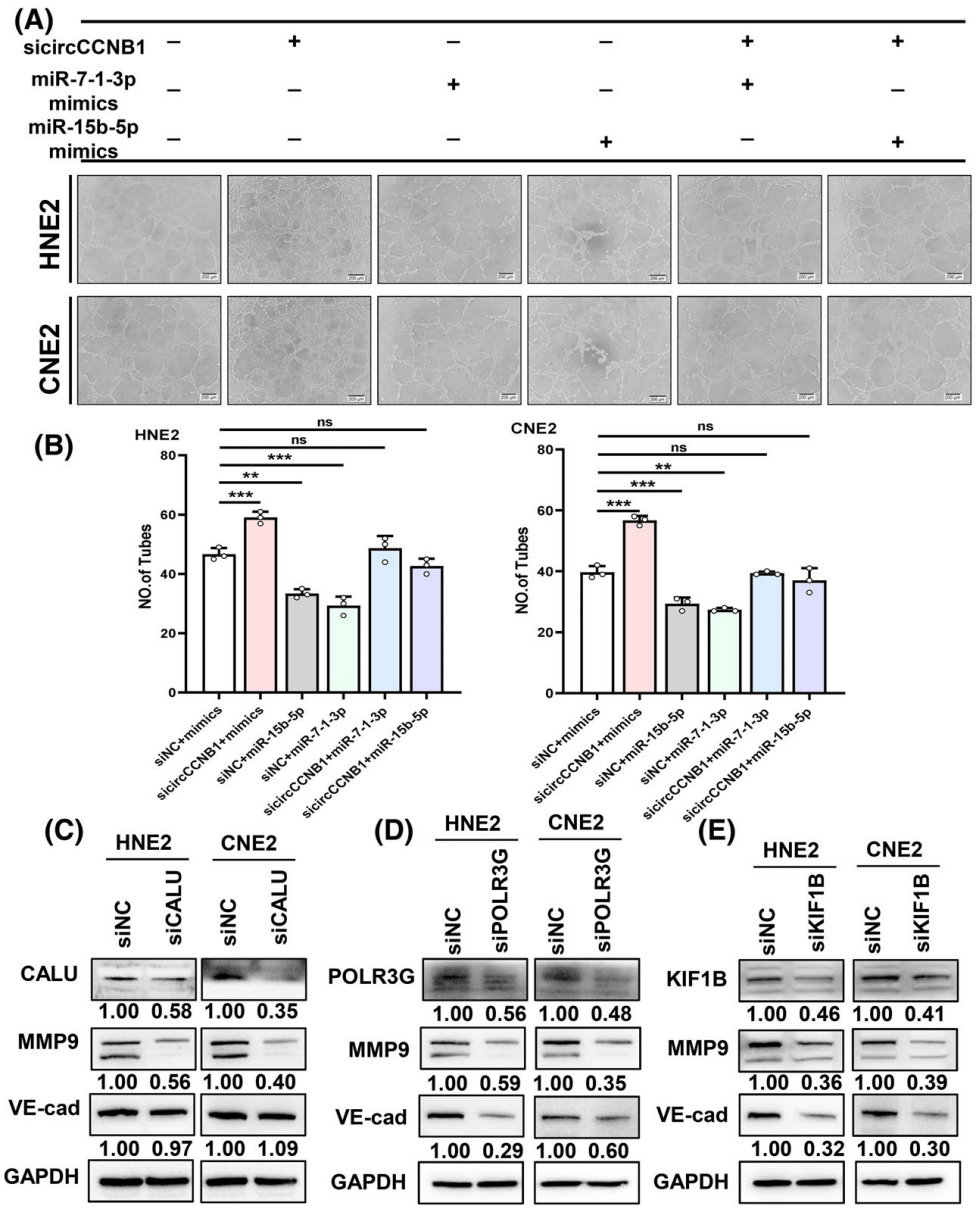
The circCCNB1‐NF90‐miR‐15b‐5p/miR‐7‐1‐3p axis inhibits the expression of CALU/KIF1B or POLR3G, thereby suppressing vasculogenic mimicry in NPC cells. (A) Co‐transfected NPC cells with sicircCCNB1 and miR‐15b‐5p/miR‐7‐1‐3p mimics, tube formation assay was used to assess VM. Scale bars = 200 μm. (B) Quantification of (A) was performed using imagej. (C–E) Western blotting was used to test the protein expression of MMP9/VE‐cadherin after transfected NPC cells with siCALU/siKIF1B/siPOLR3G. VM, vasculogenic mimicry. The numbers below the blots indicate the value of signal intensity scanned by imagej software (normalized to corresponding GAPDH bands). Unpaired two‐tailed Student's *t* test was used, **P < 0.01, ***P < 0.001; ns, not significant. Results for (A) were derived from three independent repetitions. Western blots were reproduced three times with similar results for (C–E). Data are presented as mean ± SD.

In order to explore the effects of miR‐15b‐5p and miR‐7‐1‐3p on tube formation ability, we transfected mimics or inhibitors of miR‐15b‐5p and miR‐7‐1‐3p respectively and conducted tube formation assays. The results showed that miR‐15b‐5p and miR‐7‐1‐3p inhibit VM in NPC (Fig. 6C, Fig. S6B). In order to prove that the effect of circCCNB1 on VM is dependent on miR‐15b‐5p and miR‐7‐1‐3p, through rescue experiment, we found that miR‐15b‐5p or miR‐7‐1‐3p mimics partially reversed the increase of tube formation caused by sicirciCCNB1 (Fig. 7A,B).

To determine whether the target gene CALU/KIF1B/POLR3G affects the VM, we knocked down CALU/KIF1B/POLR3G respectively in NPC (Fig. S6C). Moreover, through tube formation assays, it was found that knocking down CALU/KIF1B/POLR3G could inhibit the VM in NPC cells (Fig. S6D). Notably, we also found that the increase in tube formation caused by sicircCCNB1 could be partially rescued by co‐transfected with siCALU/siKIF1B/siPOLR3G and sicircCCNB1 (Fig. S7A). This indicated that circCCNB1 inhibited the VM through the CALU/KIF1B/POLR3G axis. In the process of VM, MMP2, MMP9, and VE‐cadherin are usually changed, which are also markers of VM. Subsequently, we detected whether knocking down CALU/KIF1B/POLR3G affected the expression of these markers, respectively. The results showed that siCALU could significantly inhibit the expression of MMP9 (Fig. S7B), and siPOLR3G or siKIF1B could decrease the expression of MMP9 and VE‐cadherin (Fig. S7C,D). The same effects were confirmed by western blotting (Fig. 7C–E). We checked the expression of MMP9 and VE‐cadherin (encoded by CDH5) expression in TCGA database, the results demonstrate that MMP9 and CDH5 are highly expressed in HNSC tissues (Fig. S7E).

To evaluate the correlation between key players, we used TCGA database to analyze the correlation between ILF3 (encode NF90) and the other downstream molecules, the spearman correlation analysis showed that ILF3 was positively correlated with KIF1B, POLR3G, MMP9, CALU and CDH5 (encode VE‐cadherin) (Fig. S8A), CALU and KIF1B are positively correlated with CDH5 or MMP9 (Fig. S8B). And we used CancerMIRNome (http://bioinfo.jialab‐ucr.org/CancerMIRNome/#tab‐1929‐4) to assess the correlation of miRNAs and their targets. The results showed that miR‐15b‐5p is negatively correlated with CALU, while the other two targets cannot find in this website (Fig. S8C).

## 4 Discussion

NPC is a highly malignant tumor originating from the nasopharyngeal epithelium. It has obvious regional characteristics and is highly prevalent in Southeast Asia, especially in southern China. Studies have shown that recurrence and metastasis are the main reasons for treatment failure of NPC. We previously found that circCCNB1 inhibited the migration and invasion of NPC by binding to NF90. CircCCNB1 has also been studied in other tumors. Xinguang Liu et al. found that in hepatocellular carcinoma, circCCNB1 acts as a miRNA sponge to competitively adsorb miR‐106b‐5p, promotes the downstream GPM6A expression, inhibits the expression of DYNC1I1 and the AKT/ERK pathway, thereby inhibiting tumor cell proliferation [27]. Zhou et al. reported that circCCNB1 promotes CCND1 mRNA levels by binding to miR‐516b‐5p and HuR, thereby promoting glioma cell proliferation and cell cycle process [28]. However, except for our previous report, there have been no other reports on the function of circCCNB1 in NPC. By reviewing the literature on the function of NF90, it was reported that NF90 may negatively regulate the processing of miRNAs. Therefore, we speculated that circCCNB1 may affect the processing of miRNAs by binding to NF90. Our subsequent experiments indeed confirmed that circCCNB1 competitively binds to NF90, preventing it from binding to pri‐miR‐15b‐5p and pri‐miR‐7‐1‐3p, thereby increasing the chance of DGCR8 binding to pri‐miRNAs and promoting mature miRNA processing.

Considering that NF90 can serve as a microprocessor‐related protein, and regulate the splicing and processing of miRNAs. In this study, we confirmed that NF90 does not bind to microprocessor DGCR8 and Drosha directly in NPC (Fig. S3B). In addition, the NF90 and NF45 complexes bind to endogenous pri‐let‐7a in a manner that is independent of the microprocessor complex and inhibit its processing and production [29]. NF90/NF110 inhibits the processing of pri‐miR‐3173 by binding to pri‐miR‐3173, and inhibits microprocessors binding to pri‐miR‐3173 [30]. These two studies indicate that NF90 may form a complex with NF45 or NF110 to regulate the processing and production of miRNAs. However, the latest research shows that NF90 can also act alone as a negative regulator of miRNA processing, regulating the processing and generation of a type of miRNAs. This study found that circCCNB1 can competitively bind to NF90, preventing it from binding to pri‐miRNAs, thus promoting the processing of pri‐miRNAs by DGCR8. However, whether NF90 acts alone as a negative regulator of miRNA processing or through the NF110/NF45 complex, more research is needed.

During anti‐angiogenic treatment, even though endothelium‐derived neo‐angiogenesis is under suppression, the tumor growth would alternatively dependent on the supplies from VM. Developing strategies against VM formation would be a promising therapeutic regimen for solid tumors. There were few reports about circRNAs regulate VM formation. For example, tyrosine kinase inhibitor (TKI)‐induced Erβ up‐regulates circDGKD transcriptionally, which enhances VE‐cadherin expression by sponging miR‐125‐5p, thus inducing VM formation of renal cell carcinoma [31]. Androgen receptor (AR) could directly target the circR7 host gene promoter to suppress circR7 formation, up‐regulate miR‐7‐5p, which might directly target the VE‐cadherin and Notch4 3′UTR to suppress VM formation in hepatocellular carcinoma [32]. Knockdown of circRNA ZNF292 (cZNF292) increased SOX9 nuclear translocation, subsequently reduced Wnt/β‐catenin pathway activity, leading to suppression of VM in hypoxic hepatoma cell [33]. But in NPC, there is only one reference showed that circRNA could affect VM activity: circMAN1A2 promotes VM formation of NPC via miR‐940/ERBB2 axis and further activates the PI3K/AKT/mTOR pathway [34]. The complex role of circRNAs in VM of NPC cells has not yet been elucidated.

According to previous research, miR‐15b‐5p/miR‐7‐1‐3p and their target genes are related to angiogenesis. NF90 has been reported to participate in the angiogenesis of endothelial cells [25], but whether NF90 is involved in the VM of tumor cells has not yet been reported. Although there are many anti‐angiogenic drugs, their clinical efficacy are still unsatisfactory, VM‐mediated new blood vessels may be an important reason for the failure. Therefore, this study mainly focused on explore whether circCCNB1‐NF90‐miRNAs affected the VM of NPC. To further confirm that the circCCNB1‐NF90‐miR‐15b‐5p/miR‐7‐1‐3p axis is related to VM, we detected changes of VM markers and found that the target gene of miR‐15b‐5p/miR‐7‐1‐3p, KIF1B/CALU/POLR3G, could positively regulate the expression of MMP9 and VE‐cadherin. This is an important discovery of the VM regulatory mechanism in NPC.

## 5 Conclusions

**Fig. 8:**
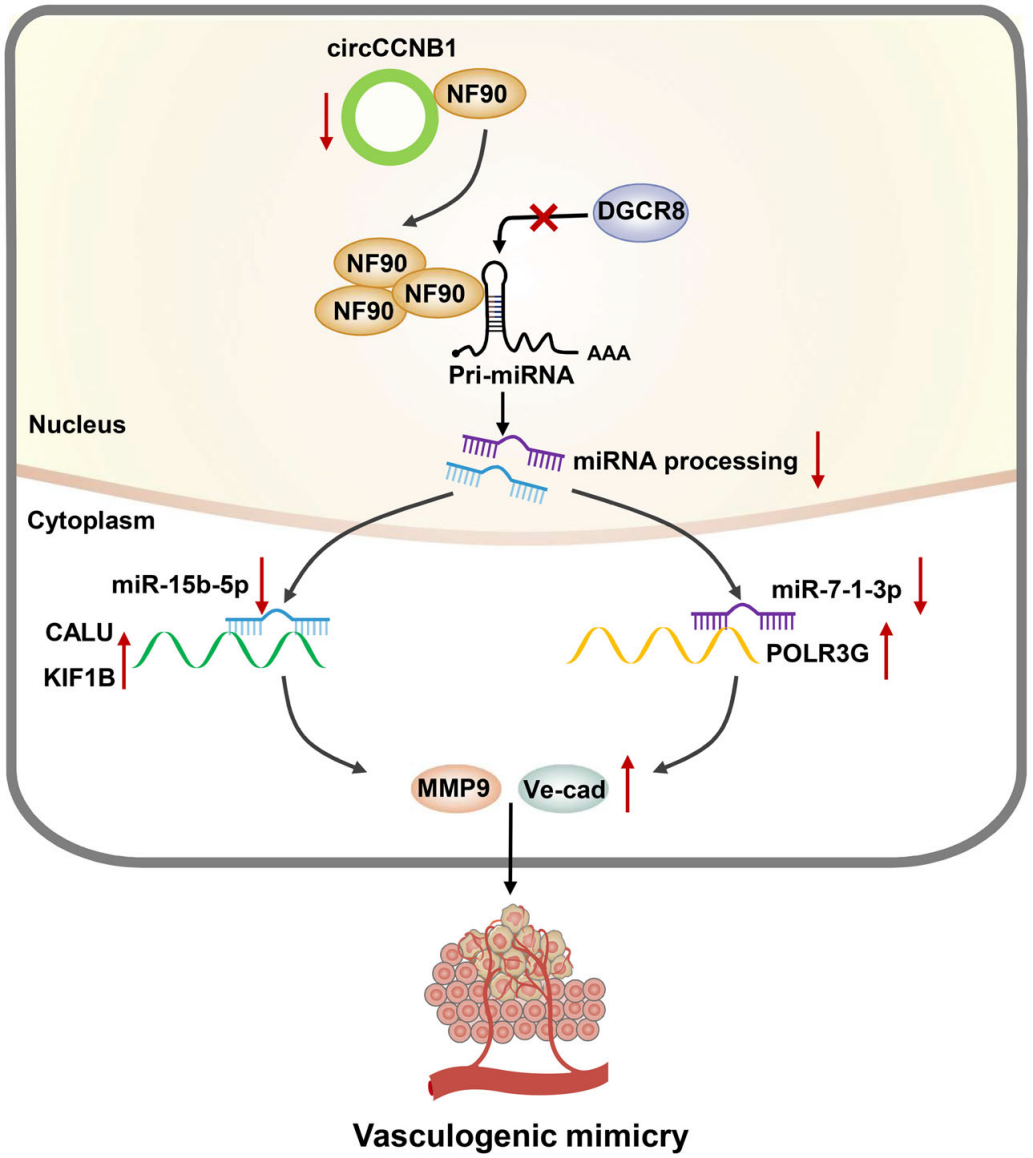
Working model. CircCCNB1 is down‐regulated in NPC and binds less NF90, more NF90 binds to pri‐miRNAs, thus blocking the chance of DGCR8 binding to pri‐miRNAs and inhibiting the processing and generation of miR‐15b‐5p/miR‐7‐1‐3p. Furthermore, decreased miR‐15b‐5p/miR‐7‐1‐3p promotes the expression of target genes KIF1B/CALU/POLR3G, thus elevating the expression of MMP9 and VE‐cadherin to promote the vasculogenic mimicry in NPC.

In summary, we discovered a new VM pathway mediated by circCCNB1. Mechanistically, we found that circCCNB1 competitively binds to NF90, inhibits the binding of NF90 to pri‐miR‐7‐1 and pri‐miR‐15b, thus enabling more pri‐miRNAs to bind to microprocessor DGCR8, and promoting the processing of mature miR‐7‐1‐3p and miR‐15b‐5p. The up‐regulation of mature miR‐7‐1‐3p and miR‐15b‐5p inhibits the expression of target genes POLR3G, CALU, and KIF1B, further decreases the expression of VM‐related genes MMP9 and VE‐cadherin, and ultimately decreasing VM of NPC cells (Fig. 8). CircCCNB1 may provide additional benefits for anti‐angiogenic therapy or combined therapies in NPC.

## Conflict of interest

The authors declare no conflict of interest.

## Author contributions

CF and FT contributed to conducting experiments, data analysis, drafting and editing the manuscript. WG, HH and WX designed, revised, and finalized the manuscript. JW and ZZ contributed to designing and revising. All authors agreed to submit.

## Supporting information


**Fig S1.** The expression level of NF90, hsa‐miR‐15b‐5p and hsa‐miR‐7‐1‐3p.
**Fig. S2.** CircCCNB1 binds to but not regulates NF90.
**Fig. S3.** CircCCNB1 inhibits pri‐miR‐15b and pri‐miR‐7‐1 but promotes mature miR‐15b‐5p and miR‐7‐1‐3p levels.
**Fig. S4.** miR‐15b‐5p targets KIF1B and CALU, while miR‐7‐1‐3p targets POLR3G.
**Fig. S5.** CALU, KIF1B and POLR3G expression in TCGA database and their binding abilities between target miRNAs.
**Fig. S6.** The effects of circCCNB1 and its downstream genes on vasculogenic mimicry.
**Fig. S7.** The effects of CALU, KIF1B and POLR3G on MMP9 and VE‐cadherin.
**Fig. S8.** Correlation among the key molecules.

**Table S1.** siRNA sequences for target genes.
**Table S2.** Primers for qRT‐PCR.
**Table S3.** Antibodies catalog information.
**Table S4.** Primer for deletion mutant and synthetic sequences for luciferase plasmids.
**Table S5.** Predicted pri‐miRNAs bind to NF90 in eCLIP data ENCSR786USC.

## Data Availability

The authors declared that all the data were available on line or from the corresponding author on reasonable request.

## References

[1] Fan C , Tang Y , Wang J , Wang Y , Xiong F , Zhang S , et al. Long non‐coding RNA LOC284454 promotes migration and invasion of nasopharyngeal carcinoma via modulating the rho/Rac signaling pathway. Carcinogenesis. 2019;40:380–391. 30380023 10.1093/carcin/bgy143

[2] Fan C , Qu H , Xiong F , Tang Y , Tang T , Zhang L , et al. CircARHGAP12 promotes nasopharyngeal carcinoma migration and invasion via ezrin‐mediated cytoskeletal remodeling. Cancer Lett. 2020;496:41–56. 32931883 10.1016/j.canlet.2020.09.006

[3] Xu R , Ding P , Zhao X , Li Z , Liu F , Gu L , et al. Circular RNA circ‐TNRC6B inhibits the proliferation and invasion of esophageal squamous cell carcinoma cells by regulating the miR‐452‐5p/DAG1 axis. Mol Oncol. 2023;17:1437–1452. 37014625 10.1002/1878-0261.13432 PMC10323880

[4] Liu A , Jiang B , Song C , Zhong Q , Mo Y , Yang R , et al. Isoliquiritigenin inhibits circ0030018 to suppress glioma tumorigenesis via the miR‐1236/HER2 signaling pathway. MedComm (2020). 2023;4:e282. 37250146 10.1002/mco2.282 PMC10220153

[5] Zhan Y , Liu Y , Yang R , Chen Q , Teng F , Huang Y , et al. CircPTEN suppresses human clear cell renal carcinoma progression and resistance to mTOR inhibitors by targeting epigenetic modification. Drug Resist Updat. 2023;71:101003. 37866104 10.1016/j.drup.2023.101003

[6] Guo X , Gao C , Yang DH , Li S . Exosomal circular RNAs: a chief culprit in cancer chemotherapy resistance. Drug Resist Updat. 2023;67:100937. 36753923 10.1016/j.drup.2023.100937

[7] Xie J , Ye F , Deng X , Tang Y , Liang JY , Huang X , et al. Circular RNA: a promising new star of vaccine. J Transl Int Med. 2023;11:372–381. 38130633 10.2478/jtim-2023-0122 PMC10732498

[8] Zhao M , Wang Y , Tan F , Liu L , Hou X , Fan C , et al. Circular RNA circCCNB1 inhibits the migration and invasion of nasopharyngeal carcinoma through binding and stabilizing TJP1 mRNA. Sci China Life Sci. 2022;65:2233–2247. 35471687 10.1007/s11427-021-2089-8

[9] Folberg R , Hendrix MJ , Maniotis AJ . Vasculogenic mimicry and tumor angiogenesis. Am J Pathol. 2000;156:361–381. 10666364 10.1016/S0002-9440(10)64739-6 PMC1850026

[10] Jiang X , Wang J , Deng X , Xiong F , Zhang S , Gong Z , et al. The role of microenvironment in tumor angiogenesis. J Exp Clin Cancer Res. 2020;39:204. 32993787 10.1186/s13046-020-01709-5 PMC7526376

[11] Grasso G , Higuchi T , Mac V , Barbier J , Helsmoortel M , Lorenzi C , et al. NF90 modulates processing of a subset of human pri‐miRNAs. Nucleic Acids Res. 2020;48:6874–6888. 32427329 10.1093/nar/gkaa386 PMC7337520

[12] Chen G , Yang Y , Wu QJ , Cao L , Ruan W , Shao C , et al. ILF3 represses repeat‐derived microRNAs targeting RIG‐I mediated type I interferon response. J Mol Biol. 2022;434:167469. 35120969 10.1016/j.jmb.2022.167469

[13] Liu CX , Li X , Nan F , Jiang S , Gao X , Guo SK , et al. Structure and degradation of circular RNAs regulate PKR activation in innate immunity. Cell. 2019;177:865–880.e21. 31031002 10.1016/j.cell.2019.03.046

[14] Li Y , Xue JY , Chen S , Wang C , Sun P , Fu S , et al. LncRNA PVT1 is a novel mediator promoting the angiogenesis response associated with collateral artery formation. Int J Biochem Cell Biol. 2022;151:106294. 36041701 10.1016/j.biocel.2022.106294

[15] Bhagavatheeswaran S , Ramachandran V , Shanmugam S , Balakrishnan A . Isopimpinellin extends antiangiogenic effect through overexpression of miR‐15b‐5p and downregulating angiogenic stimulators. Mol Biol Rep. 2022;49:279–291. 34709570 10.1007/s11033-021-06870-4

[16] Bai Y , Gong X , Dong R , Cao Z , Dou C , Liu C , et al. Long non‐coding RNA HCAR promotes endochondral bone repair by upregulating VEGF and MMP13 in hypertrophic chondrocyte through sponging miR‐15b‐5p. Genes Dis. 2022;9:456–465. 35224160 10.1016/j.gendis.2020.07.013 PMC8843884

[17] Zhu LP , Zhou JP , Zhang JX , Wang JY , Wang ZY , Pan M , et al. MiR‐15b‐5p regulates collateral artery formation by targeting AKT3 (protein kinase B‐3). Arterioscler Thromb Vasc Biol. 2017;37:957–968. 28254819 10.1161/ATVBAHA.116.308905

[18] Chen S , Han M , Chen W , He Y , Huang B , Zhao P , et al. KIF1B promotes glioma migration and invasion via cell surface localization of MT1‐MMP. Oncol Rep. 2016;35:971–977. 26576027 10.3892/or.2015.4426

[19] Dong Z , Xu X , Du L , Yang Y , Cheng H , Zhang X , et al. Leptin‐mediated regulation of MT1‐MMP localization is KIF1B dependent and enhances gastric cancer cell invasion. Carcinogenesis. 2013;34:974–983. 23354307 10.1093/carcin/bgt028

[20] Wang Q , Shen B , Chen L , Zheng P , Feng H , Hao Q , et al. Extracellular calumenin suppresses ERK1/2 signaling and cell migration by protecting fibulin‐1 from MMP‐13‐mediated proteolysis. Oncogene. 2015;34:1006–1018. 24632605 10.1038/onc.2014.52

[21] Renaud M , Praz V , Vieu E , Florens L , Washburn MP , l'Hote P , et al. Gene duplication and neofunctionalization: POLR3G and POLR3GL. Genome Res. 2014;24:37–51. 24107381 10.1101/gr.161570.113 PMC3875860

[22] Park CR , Lee M , Lee SY , Kang D , Park SJ , Lee DC , et al. Regulating POLR3G by microRNA‐26a‐5p as a promising therapeutic target of lung cancer stemness and chemosensitivity. Noncoding RNA Res. 2023;8:273–281. 36949748 10.1016/j.ncrna.2023.03.001 PMC10025963

[23] Lautre W , Richard E , Feugeas JP , Dumay‐Odelot H , Teichmann M . The POLR3G subunit of human RNA polymerase III regulates tumorigenesis and metastasis in triple‐negative breast cancer. Cancers (Basel). 2022;14(23):5732. 10.3390/cancers14235732 36497214 PMC9735567

[24] Dunleavey JM , Xiao L , Thompson J , Kim MM , Shields JM , Shelton SE , et al. Vascular channels formed by subpopulations of PECAM1+ melanoma cells. Nat Commun. 2014;5:5200. 25335460 10.1038/ncomms6200 PMC4261234

[25] Zhang W , Xiong Z , Wei T , Li Q , Tan Y , Ling L , et al. Nuclear factor 90 promotes angiogenesis by regulating HIF‐1alpha/VEGF‐A expression through the PI3K/Akt signaling pathway in human cervical cancer. Cell Death Dis. 2018;9:276. 29449553 10.1038/s41419-018-0334-2 PMC5833414

[26] Zhou Q , Zhu Y , Wei X , Zhou J , Chang L , Sui H , et al. MiR‐590‐5p inhibits colorectal cancer angiogenesis and metastasis by regulating nuclear factor 90/vascular endothelial growth factor A axis. Cell Death Dis. 2016;7:e2413. 27735951 10.1038/cddis.2016.306 PMC5133975

[27] Liu YM , Cao Y , Zhao PS , Wu LY , Lu YM , Wang YL , et al. CircCCNB1 silencing acting as a miR‐106b‐5p sponge inhibited GPM6A expression to promote HCC progression by enhancing DYNC1I1 expression and activating the AKT/ERK signaling pathway. Int J Biol Sci. 2022;18:637–651. 35002514 10.7150/ijbs.66915 PMC8741844

[28] Li X , Wang C , Chen G , Zou W , Deng Y , Zhou F . EIF4A3‐induced circCCNB1 (hsa_circ_0001495) promotes glioma progression by elevating CCND1 through interacting miR‐516b‐5p and HuR. Metab Brain Dis. 2022;37:819–833. 35038081 10.1007/s11011-021-00899-x

[29] Haselmann V , Kurz A , Bertsch U , Hubner S , Olempska‐Muller M , Fritsch J , et al. Nuclear death receptor TRAIL‐R2 inhibits maturation of let‐7 and promotes proliferation of pancreatic and other tumor cells. Gastroenterology. 2014;146:278–290. 24120475 10.1053/j.gastro.2013.10.009

[30] Barbier J , Chen X , Sanchez G , Cai M , Helsmoortel M , Higuchi T , et al. An NF90/NF110‐mediated feedback amplification loop regulates dicer expression and controls ovarian carcinoma progression. Cell Res. 2018;28:556–571. 29563539 10.1038/s41422-018-0016-8 PMC5951825

[31] Ding J , Cui XG , Chen HJ , Sun Y , Yu WW , Luo J , et al. Targeting circDGKD intercepts TKI's effects on up‐regulation of estrogen receptor beta and vasculogenic mimicry in renal cell carcinoma. Cancers (Basel). 2022;14:1639. 35406411 10.3390/cancers14071639 PMC8996923

[32] Bao S , Jin S , Wang C , Tu P , Hu K , Lu J . Androgen receptor suppresses vasculogenic mimicry in hepatocellular carcinoma via circRNA7/miRNA7‐5p/VE‐cadherin/Notch4 signalling. J Cell Mol Med. 2020;24:14110–14120. 33118329 10.1111/jcmm.16022 PMC7754040

[33] Yang W , Liu Y , Gao R , Xiu Z , Sun T . Knockdown of cZNF292 suppressed hypoxic human hepatoma SMMC7721 cell proliferation, vasculogenic mimicry, and radioresistance. Cell Signal. 2019;60:122–135. 31028816 10.1016/j.cellsig.2019.04.011

[34] Mo H , Shen J , Zhong Y , Chen Z , Wu T , Lv Y , et al. CircMAN1A2 promotes vasculogenic mimicry of nasopharyngeal carcinoma cells through upregulating ERBB2 via sponging miR‐940. Oncol Res. 2022;30:187–199. 37304410 10.32604/or.2022.027534 PMC10208074

