# Supplementary material for: CircCCNB1 inhibits vasculogenic mimicry by sequestering NF90 to promote miR‐15b‐5p and miR‐7‐1‐3p processing in nasopharyngeal carcinoma
Source: Mol Oncol. 2025 Feb 18;19(6):1876–93. doi: 10.1002/1878-0261.13821 (PMC12161461; doi:10.1002/1878-0261.13821)

Fig. S2

A

Protein coding potential

**IRES Elements**	Parameter Index		
	**Position (start--end)**	**R Score**	**With Pseudoknot (Y/N)**
	182--249	1.497414	Y
	5--31	1.372278	Y
**Open Reading Frame (ORF)**	No open reading frame was found ! (Protein length less than 100aa)		
**Protein Features**	The possibility of encoding protein is relatively low(R<1.6 or it has no open reading frame), so no protein features was predicted!		

B

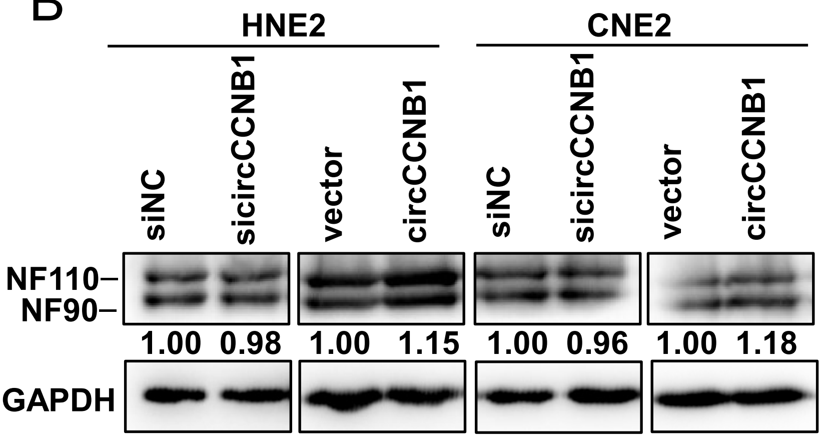

C

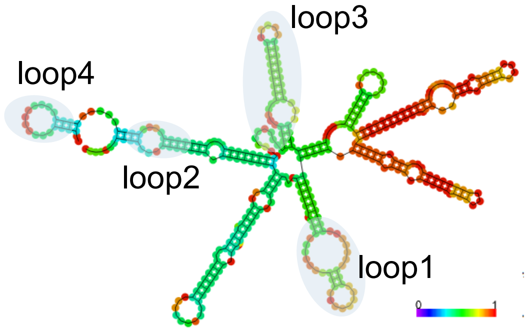

D

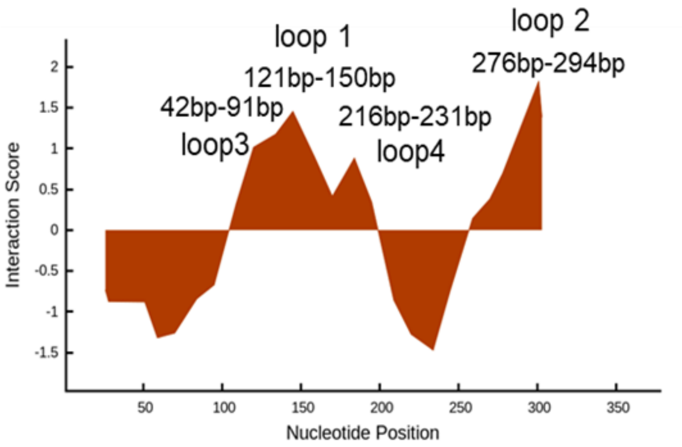

E

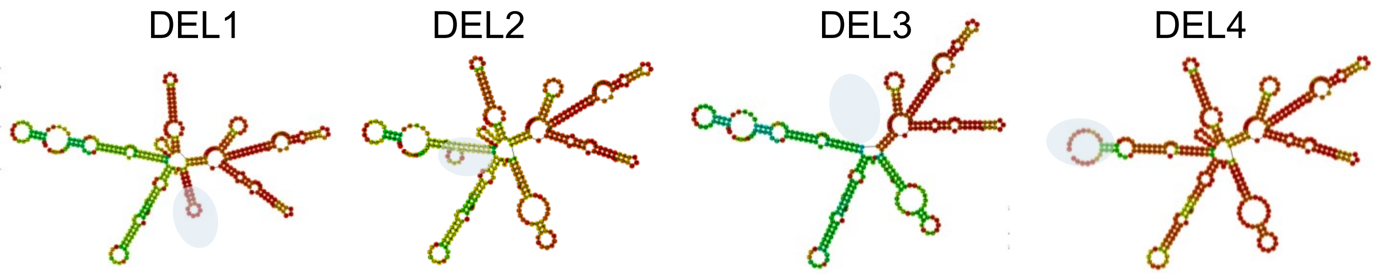

Supplement: Supplementary file 1 — Fig S1. The expression level of NF90, hsa‐miR‐15b‐5p and hsa‐miR‐7‐1‐3p. Fig. S2. CircCCNB1 binds to but not regulates NF90. Fig. S3. CircCCNB1 inhibits pri‐miR‐15b and pri‐miR‐7‐1 but promotes mature miR‐15b‐5p and miR‐7‐1‐3p levels. Fig. S4. miR‐15b‐5p targets KIF1B and CALU, while miR‐7‐1‐3p targets POLR3G. Fig. S5. CALU, KIF1B and POLR3G expression in TCGA database and their binding abilities between target miRNAs. Fig. S6. The effects of circCCNB1 and its downstream genes on vasculogenic mimicry. Fig. S7. The effects of CALU, KIF1B and POLR3G on MMP9 and VE‐cadherin. Fig. S8. Correlation among the key molecules. [file MOL2-19-1876-s001.zip › mol213821-sup-0001-FigsS1-S8/FigS2.pdf]
