# Supplementary material for: CircCCNB1 inhibits vasculogenic mimicry by sequestering NF90 to promote miR‐15b‐5p and miR‐7‐1‐3p processing in nasopharyngeal carcinoma
Source: Mol Oncol. 2025 Feb 18;19(6):1876–93. doi: 10.1002/1878-0261.13821 (PMC12161461; doi:10.1002/1878-0261.13821)
Supplement: Supplementary file 1 — Fig S1. The expression level of NF90, hsa‐miR‐15b‐5p and hsa‐miR‐7‐1‐3p. Fig. S2. CircCCNB1 binds to but not regulates NF90. Fig. S3. CircCCNB1 inhibits pri‐miR‐15b and pri‐miR‐7‐1 but promotes mature miR‐15b‐5p and miR‐7‐1‐3p levels. Fig. S4. miR‐15b‐5p targets KIF1B and CALU, while miR‐7‐1‐3p targets POLR3G. Fig. S5. CALU, KIF1B and POLR3G expression in TCGA database and their binding abilities between target miRNAs. Fig. S6. The effects of circCCNB1 and its downstream genes on vasculogenic mimicry. Fig. S7. The effects of CALU, KIF1B and POLR3G on MMP9 and VE‐cadherin. Fig. S8. Correlation among the key molecules. [file MOL2-19-1876-s001.zip › mol213821-sup-0001-FigsS1-S8/FigsS1-S8_Legends.docx]

**Supporting Information**

**Supplementary Figure**

**Fig S1 The expression level of NF90, hsa-miR-15b-5p and hsa-miR-7-1-3p**

(A). ILF3 expression in normal and head and neck squamous carcinoma (HNSC) was analyzed using TCGA database. (B). Expression of hsa-miR-15b-5p and hsa-miR-7-1-3p in NPC tissues and normal nasopharyngeal epithelium (NPE) was analyzed in microarray GSE32960. (C). Secondary structures of pri-miR-15b and pri-miR-7-1 were predicted using RNAfold, with pri-miR-200a as a negative control. (D-E). qRT-PCR and western blotting were performed to measure the expression of NF90 after NF90 overexpression or knockdown. Unpaired two-tailed Student’s *t* test was used, **p < 0.01, ***p < 0.001, and ****p < 0.0001. Results for (D) were derived from three independent repetitions. Western blots were reproduced three times with similar results for (E). Data are presented as mean ± SD.

**Fig S2 CircCCNB1 binds to but not regulates NF90**
(A). The translation potential of circCCNB1 was predicted by circRNADb (<http://reprod.njmu.edu.cn/cgi-bin/circrnadb/circRNADb.php>). (B). NF90 expression was detected using western blotting after cells transfected with sicircCCNB1 or circCCNB1 overexpression vectors (C). The secondary structure of circCCNB1 was predicted using RNAfold. (D). The specific binding sites between circCCNB1 and NF90 were prediction using CatRAPID database. (E). The stem-loop structures of circCCNB1 after deletion of four different stem-loop sequences were predicted using RNAfold. Western blots were reproduced three times with similar results for (B).

**Fig S3 CircCCNB1 inhibits pri-miR-15b and pri-miR-7-1 but promotes mature miR-15b-5p and miR-7-1-3p levels**

(A). RNA pulldown assay was performed to examine the binding of circCCNB1 with the microprocessor proteins Drosha and DGCR8. (B). Co-immunoprecipitation was used to determine the binding of NF90 with the microprocessor proteins Drosha and DGCR8. (C). Ribonucleoprotein immunoprecipitation assays were conducted to assess the binding of DGCR8 to pri-miR-7-1 after overexpression or knockdown of circCCNB1. (D). Ribonucleoprotein immunoprecipitation assays were performed to evaluate the binding of DGCR8 to pri-miR-7-1 after overexpression of circCCNB1 and circCCNB1 DEL1 mutant. (E-F). qRT-PCR was used to measure the expression of pri-miR-15b and pri-miR-7-1 or mature miR-15b-5p and miR-7-1-3p after overexpression of circCCNB1 and circCCNB1 DEL1. Unpaired two-tailed Student’s *t* test was used, *p < 0.05, **p < 0.01, ***p < 0.001, ns: not significant. Western blots were reproduced three times with similar results for (A, B). Results for (C-F) were derived from three independent repetitions. Data are presented as mean ± SD.

**Fig S4 miR-15b-5p targets KIF1B and CALU, while miR-7-1-3p targets POLR3G**

(A-B). The target genes of miR-15b-5p or miR-7-1-3p were predicted using miRDIP, Targetscan, miRdb, and miRwalk software, and intersected with the upregulated mRNAs from the NPC datasets GSE12452 and GSE53819. (C-D). qRT-PCR was performed to measure the expression of downstream genes in HNE2 cells transfected with miR-15b-5p or miR-7-1-3p mimic/inhibitor. (E). Western blotting was conducted to examine the expression of CALU and KIF1B in NPC cells transfected with miR-15b-5p mimic or inhibitor. (F). Western blotting was conducted to examine the expression of POLR3G in NPC cells transfected with miR-7-1-3p mimic or inhibitor. Unpaired two-tailed Student’s *t* test was used, *p < 0.05, **p < 0.01, ns: not significant. Results for (C, D) were derived from three independent repetitions. Western blots were reproduced three times with similar results for (E, F). Data are presented as mean ± SD.

**Fig S5 CALU, KIF1B and POLR3G expression in TCGA database and their binding abilities between target miRNAs.**

(A). CALU, KIF1B and POLR3G expression in normal and head and neck squamous carcinoma (HNSC) was analyzed using TCGA database. (B). CALU, KIF1B and POLR3G expression in NPC tissues and normal nasopharyngeal epithelium (NPE) was analyzed using NPC microarray GSE53819. (C-D). The binding ability between miR-15b-5p and CALU or KIF1B was predicted using the RNAhybrid website, considering the minimum free energy and complementary base pairing, along with a schematic diagram illustrating the construction of wild-type and mutant vectors.
(E). The binding ability between miR-7-1-3p and POLR3G was predicted using the RNAhybrid website.

**Fig S6 The effects of circCCNB1 and its downstream genes on vasculogenic mimicry**

(A). By co-transfection of circCCNB1 and NF90, the tube formation ability was detected by tube formation assay. Scale bars=200 μm. (B). CNE2 cells transfected with miR-15b-5p/miR-7-1-3p mimics or inhibitors, tube formation assay was employed to evaluate VM. Scale bars=200 μm. (C). qRT-PCR was employed to test the RNA levels after knocking down CALU, KIF1B, POLR3G. (D). Tube formation assay was used to test VM after transfected NPC cells with siCALU, siKIF1B, siPOLR3G. Scale bars=200 μm. VM: vasculogenic mimicry. Unpaired two-tailed Student’s t test was used, *p < 0.05, **p < 0.01, ***p < 0.001, and ****p < 0.0001, ns: not significant. These experiments were derived from three independent repetitions. Data are presented as mean ± SD.

**Fig S7 The effects of CALU, KIF1B and POLR3G on MMP9 and VE-cadherin**

(A). Tube formation assay was used to test vasculogenic mimicry after co-transfected NPC cells with sicircCCNB1 and siCALU/siKIF1B/siPOLR3G. Scale bars=200 μm. (B-D). qRT-PCR was employed to test the RNA levels of MMP2/MMP9/VE-cadherin after transfected NPC cells with siCALU/siKIF1B/siPOLR3G. (E). MMP9, CDH5 expression in HNSC was analyzed using TCGA database. Unpaired two-tailed Student’s t test was used, *p < 0.05, **p < 0.01, ***p < 0.001, ns: not significant. These experiments were derived from three independent repetitions. Data are presented as mean ± SD.

**Fig S8 Correlation among the key molecules**

(A). Spearman correlation analysis was performed to evaluate the correlation between ILF3 and other molecules using HNSC samples from TCGA database (R>0, positively correlated, p<0.05, significant). (B). Spearman correlation analysis was performed to evaluate the correlation between CALU or KIF1B and downstream MMP9 and CDH5 using HNSC samples from TCGA database (rho>0, positively correlated, p<0.05, significant). (C). Spearman correlation analysis was performed to evaluate the correlation between miR-15b-5p and CALU using HNSC samples from TCGA database (R<0, negatively correlated, p<0.05, significant).

**Supplementary Table**

Table 1. siRNA sequences for target genes

Table 2. Primers for qRT-PCR

Table 3. Antibodies catalog information.

Table 4. Primer for deletion mutant and synthetic sequences for luciferase plasmids

Table 5. Predicted pri-miRNAs bind to NF90 in eCLIP data ENCSR786USC
