# Supplementary material for: CircCCNB1 inhibits vasculogenic mimicry by sequestering NF90 to promote miR‐15b‐5p and miR‐7‐1‐3p processing in nasopharyngeal carcinoma
Source: Mol Oncol. 2025 Feb 18;19(6):1876–93. doi: 10.1002/1878-0261.13821 (PMC12161461; doi:10.1002/1878-0261.13821)
Supplement: Supplementary file 2 — Table S1. siRNA sequences for target genes. Table S2. Primers for qRT‐PCR. Table S3. Antibodies catalog information. Table S4. Primer for deletion mutant and synthetic sequences for luciferase plasmids. Table S5. Predicted pri‐miRNAs bind to NF90 in eCLIP data ENCSR786USC. [file MOL2-19-1876-s002.docx]

**Table S1. siRNA sequences for target genes**

| **siRNAs** | **Sense (5’-3’)** | **Antisense (5’-3’)** |
| --- | --- | --- |
| si-circCCNB1 | GAUAAUGGUGAAUGGAAUATT | UAUUCCAUUCACCAUUAUCTT |
| si-NF90 | CCUGUGCUUCACUGGGUUUTT | AAACCCAGUGAAGCACAGGTT |
| si-KIF1B | GAGUGUUUCACUUUCUGGUGAUAAA | UUUAUCACCAGAAAGUGAAACGCUC |
| si-CALU  si-POLR3G | CUAUGUAUACAUACUUGAATT  GCAGAGCUAUAGAUAGAAATT | UUCAAGUAUGUAUACAUAGTT  UUUCUAUCUAUAGCUCUGCTT |

|  |
| --- |

**Table S2. Primers for qRT-PCR**

| **Targets** | **Primer sequences (5’-3’)** |
| --- | --- |
| CircCCNB1-F | TTCTCAAATTGCAGCAGGAG |
| CircCCNB1-R | CACCAATTTCTGGAGGGTACA |
| NF90-F | CTAGACAGACAGCAACGGGA |
| NF90-R | GCGTAATGGCATAGGTGGTG |
| pri-miR-7-1-F | CCATTACAGGAACACAGGACC |
| pri-miR-7-1-R | GGCAGCAGTTTTCACCAGAG |
| pri-miR-15b-F | TGTTCCACTCTAGCAGCACG |
| pri-miR-15b-R | TGCTGTATCCCTGTCACACT |
| pri-miR-200a-F | GAGCAGGACCCAACAGAGG |
| pri-miR-200a -R | CAGGAGGGAAGATGGCTGTG |
| GAPDH-F | CAAGGTCATCCATGACAACTTTG |
| GAPDH-R  POLR3G-F  POLR3G-R  CEP76-F  CEP76-R  EFNB2-F  EFNB2-R  PGAP1-F  PGAP1-R  ACVR1C-F  ACVR1C-R  PRLR-F  PRLR-R  KIF1B-F  KIF1B-R  AKT3-F  AKT3-R  CHEK1-F  CHEK1-R  ENAH-F  ENAH-R  GLS2-F  GLS2-R  AMMECR1-F  AMMECR1-R  CALU-F  CALU-R | GTCCACCACCCTGTTGCTGTAG  CACTTCGGCTGCAGAGTTTT  AGTGGGCAAATTCTGAAAG  GCTAAGCAGTGGTGGAGAGA  AGGGGCTCGTTCATAACCAA  GATGTTGGTCCAAAGTGCGT  AAGGAGAGGTTGGGGTGATG  CAACTTCGGCCTCAATGTC  TTCGTGGATGGTGAAGTCC  GGAAGATGGTGTGGGGAAGA  CTTGATCATTCCAGCCACGG  GCAAGCAGTACACCTCCATG  GAGCGTGAACCAACCAGTTT  GGAACAAAGCCAGGACAGTG  TTGGCACCAGTTGAATCAGC  GTTGGGTTCAGAAGAGGGGA  TGTGTTTGGCTTTGGTCGTT  TATGAAGCGTGCCGTAGACT  ATAAACCACCCCTGCCATGA  CTTGGTTGGTTCTGCTGACC  AGACTCACTGTGATGCCCAA  CTGCAGCTGAAGGACACATC  TGTCCATGACCTGTGCTCAT  ATGCCCAAATGCTGCTTCAA  CACACAACGGGTTTCCACTT  GCAAGCTTGGAACTGGAGAG  ACCGTAGACCCAGGAGTAGT |
| LAMC1-F | GCCTTCCTGACCGACTACAACAAC |
| LAMC1-R | GCGGCTGGTGTGGAACTTGAG |
| MMP2-F | GGATGATGCCTTTGCTCGT |
| MMP2-R | GGAGTCCGTCCTTACCGTC |
| VE-cadherin-F | GAAAAACACCTCACTTCCCCATC |
| VE-cadherin-R | CTTGCCCACATATTCTCCTTTG |
| MMP9-F | GAGAAGAGAGGGCCCAGC |
| MMP9-R | ACGTGACCTATGACATCCTGC |

**Table S3. Antibodies catalog information**

| **Item** | **Catalog number** | **Company** |
| --- | --- | --- |
| DGCR8 Rabbit mAb | 204013-T42 | Sino Biological |
| Drosha Mouse mAb | 102288-T32 | Sino Biological |
| NF90 Rabbit Antibody | 19887-1-AP | Proteintech |
| Anti-rabbit IgG, HRP-linked Antibody | 7074P2 | Cell Signaling Technology |
| Anti-mouse IgG, HRP-linked Antibody | 7076P2 | Cell Signaling Technology |
| DYKDDDDK tag (Flag) Recombinant Antibody | 80010-1-RR | Proteintech |
| CALU Rabbit Polyclonal Antibody | AWA59126 | Abiowell |
| KIF1B Polyclonal Antibody | YN4193 | Immunoway |
| POLR3G Polyclonal Antibody | YT5489 | Immunoway |
| MMP9 Polyclonal Antibody | YT1892 | Immunoway |
| VE-cadherin Mouse McAb | 66804-1-Ig | Proteintech |

**Table S4. Primer for deletion mutant and synthetic sequences for luciferase plasmids**

| **Targets** | **Sequences (5’-3’)** |
| --- | --- |
| **NF90 truncated mutant** | |
| ∆1-342-F | CGGGGTACCATGCCCAAGAAACCAAAG |
| ∆1-342-R | CGCGGATCCTTACTTATCGTCGTCATCCTTGTAATCGGAAGACCCAAAATCATGAT |
| ∆1-470-F | CGGGGTACCATG GCTGAAGGCAGGGACTCGAG |
| ∆1-470-R | CGCGGATCCTTACTTATCGTCGTCATCCTTGTAATCGGAAGACCCAAAATCATGAT |
| ∆343-702-F | CGGGGTACCATGCGTCCAATGCGAATTTT |
| ∆343-702-R | CGCGGATCCTTACTTATCGTCGTCATCCTTGTAATCCTTGGAAGGCAGAGGGTCCA |
| ∆471-702-F | CGGGGTACCATGCGTCCAATGCGAATTTT |
| ∆471-702-R | CGCGGATCCTTACTTATCGTCGTCATCCTTGTAATCACCCGTCGGCAAGCCCAT |
| **Synthetic sequences for wild type or mutant** | |
| POLR3G-wt | AACATTTGGACAGACTTGATTTGTATTTTATTTC |
| POLR3G-mt | AACATTTGGAATTTTATTTC |
| CALU-wt | GTAAACGTGTGCCATGGTGGTTTGCTGCACGAGTTTTT |
| CALU-mt | GTAAACGTGTGTTACGAGTTTTT |
| KIF1B-wt | GGGTTTGGCCGTGGGCCGTGATGGCAGCAGGCG |
| KIF1B-mt | GGGTTTGGCCGCAGCAGGCG |

**Table S5. Predicted pri-miRNAs bind to NF90 in eCLIP data ENCSR786USC**

**Predicted pri-miRNAs bind to NF90**

pri-miR-1273c pri-miR-1290 pri-miR-15b pri-miR-186

pri-miR-1914 pri-miR-3140 pri-miR-3145 pri-miR-3173

pri-miR-3189 pri-miR-3646 pri-miR-7-1 pri-miR-3680-1

pri-miR-3939 pri-miR-4485 pri-miR-4635 pri-miR-4659a

pri-miR-4687 pri-miR-4712 pri-miR-4714 pri-miR-4730

pri-miR-4762 pri-miR-4775 pri-miR-4779 pri-miR-4782

pri-miR-548aq pri-miR-548aR pri-miR-548d-1 pri-miR-548u

pri-miR-548v pri-miR-5581 pri-miR-570 pri-miR-578

pri-miR-579 pri-miR-606 pri-miR-624 pri-miR-6751

pri-miR-6839 pri-miR-3648-1
