# Supplementary material for: CircCCNB1 inhibits vasculogenic mimicry by sequestering NF90 to promote miR‐15b‐5p and miR‐7‐1‐3p processing in nasopharyngeal carcinoma
Source: Mol Oncol. 2025 Feb 18;19(6):1876–93. doi: 10.1002/1878-0261.13821 (PMC12161461; doi:10.1002/1878-0261.13821)
Supplement: Supplementary file 1 — Fig S1. The expression level of NF90, hsa‐miR‐15b‐5p and hsa‐miR‐7‐1‐3p. Fig. S2. CircCCNB1 binds to but not regulates NF90. Fig. S3. CircCCNB1 inhibits pri‐miR‐15b and pri‐miR‐7‐1 but promotes mature miR‐15b‐5p and miR‐7‐1‐3p levels. Fig. S4. miR‐15b‐5p targets KIF1B and CALU, while miR‐7‐1‐3p targets POLR3G. Fig. S5. CALU, KIF1B and POLR3G expression in TCGA database and their binding abilities between target miRNAs. Fig. S6. The effects of circCCNB1 and its downstream genes on vasculogenic mimicry. Fig. S7. The effects of CALU, KIF1B and POLR3G on MMP9 and VE‐cadherin. Fig. S8. Correlation among the key molecules. [file FigS5.pdf › mol213821-sup-0001-FigsS1-S8/FigS5.pdf]

Fig.S5

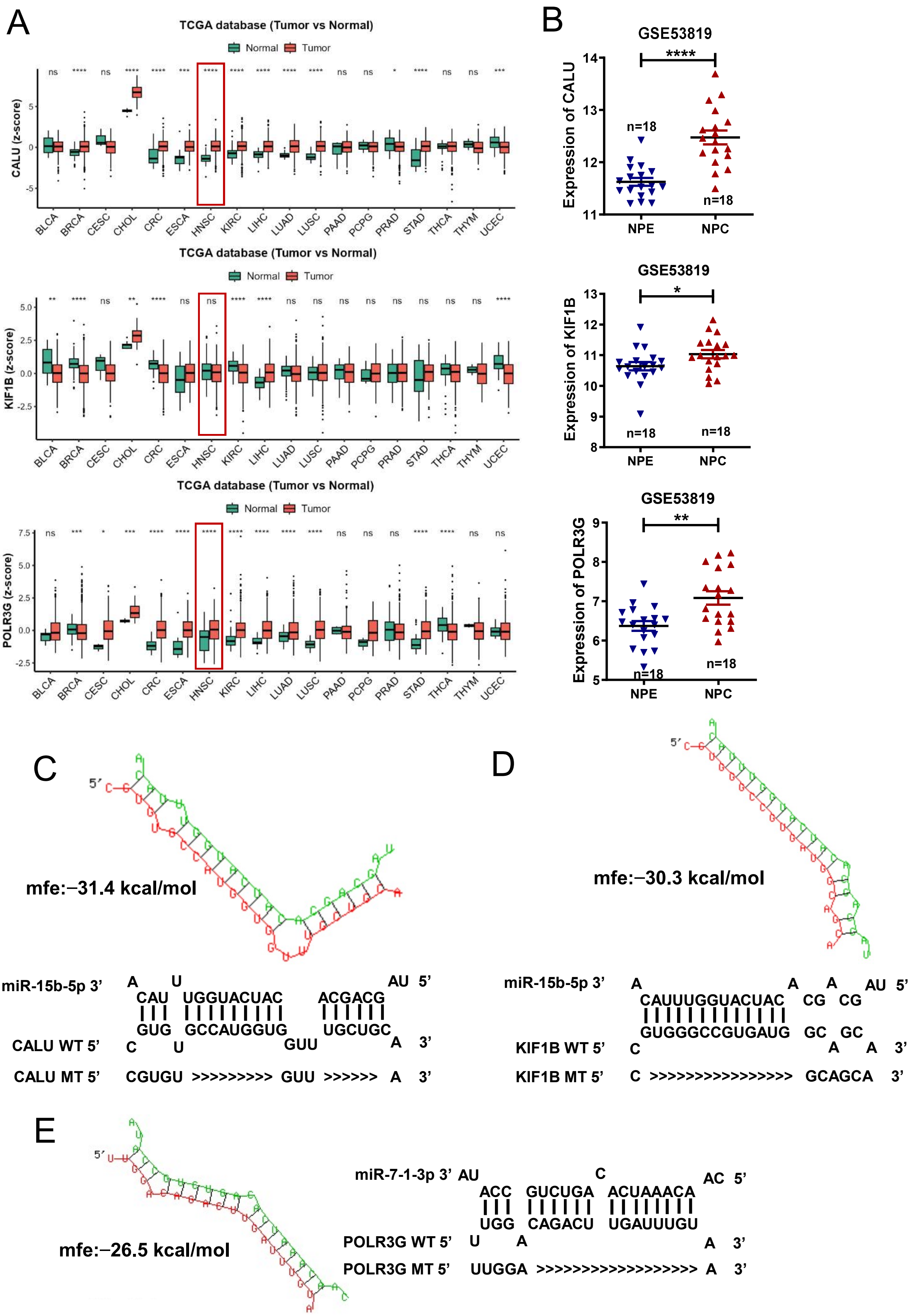

A
TCGA database (Tumor vs Normal)
Normal Tumor
CALU (z-score)
ns **** ns **** **** *** **** **** **** **** **** ns ns * **** ns ns ***
BLCA BRCA CESC CHOL CRC ESCA HNSC KIRC LIHC LUAD LUSC PAAD PCPG PRAD STAD THCA THYM UCEC
TCGA database (Tumor vs Normal)
Normal Tumor
KIF1B (z-score)
** **** ns ** **** ns ns **** **** ns ns ns ns ns ns ns ns ****
BLCA BRCA CESC CHOL CRC ESCA HNSC KIRC LIHC LUAD LUSC PAAD PCPG PRAD STAD THCA THYM UCEC
TCGA database (Tumor vs Normal)
Normal Tumor
POLR3G (z-score)
ns *** * *** **** **** **** **** **** **** **** ns ns ns **** **** ns ns
BLCA BRCA CESC CHOL CRC ESCA HNSC KIRC LIHC LUAD LUSC PAAD PCPG PRAD STAD THCA THYM UCEC
B
GSE53819
Expression of CALU
****
n=18
n=18
NPE NPC
GSE53819
Expression of KIF1B
*
n=18
n=18
NPE NPC
GSE53819
Expression of POLR3G
**
n=18
n=18
NPE NPC
C
mfe:−31.4 kcal/mol
miR-15b-5p 3' A U AU 5'
CAU UGGUACUAC ACGACG
GUG GCCAUGGUG UGCUGC
CALU WT 5' C U GUU A 3'
CALU MT 5' CGUGU >>>>>>>>> GUU >>>>>> A 3'
D
mfe:−30.3 kcal/mol
miR-15b-5p 3' A A A AU 5'
CAUUUGGUACUAC CG CG
GUGGGCCGUGAUG GC GC
KIF1B WT 5' C A A 3'
KIF1B MT 5' C >>>>>>>>>>>>>>>> GCAGCA 3'
E
mfe:−26.5 kcal/mol
miR-7-1-3p 3' AU C AC 5'
ACC GUCUGA ACUAAACA
UGG CAGACU UGAUUUGU
POLR3G WT 5' U A A 3'
POLR3G MT 5' UUGGA >>>>>>>>>>>>>>>>>> A 3'
